# The m6A modification-mediated OGDHL exerts a tumor suppressor role in ccRCC by downregulating FASN to inhibit lipid synthesis and ERK signaling

**DOI:** 10.1038/s41419-023-06090-7

**Published:** 2023-08-25

**Authors:** Jian Shi, Daojia Miao, Qingyang Lv, Keshan Wang, Qi Wang, Huageng Liang, Hongmei Yang, Zhiyong Xiong, Xiaoping Zhang

**Affiliations:** 1 Department of Urology, Union Hospital, Tongji Medical College, Huazhong University of Science and Technology, Wuhan, 430022 Hubei P.R. China; 2 Institute of Urology, Union Hospital, Tongji Medical College, Huazhong University of Science and Technology, Wuhan, 430022 Hubei P.R. China; 3 Department of Pathogenic Biology, School of Basic Medicine, Huazhong University of Science and Technology, Wuhan, 430030 Hubei P.R. China

**Keywords:** Mechanisms of disease, Cancer metabolism, Cell growth, Cell invasion, Renal cell carcinoma

## Abstract

Metabolic reprogramming is a hallmark of cancer, and the impact of lipid metabolism as a crucial aspect of metabolic reprogramming on clear cell renal cell carcinoma (ccRCC) progression has been established. However, the regulatory mechanisms underlying the relationship between metabolic abnormalities and ccRCC progression remain unclear. Therefore, this study aimed to identify key regulatory factors of metabolic reprogramming in ccRCC and provide potential therapeutic targets for ccRCC patients. Potential metabolic regulatory factors in ccRCC were screened using bioinformatics analysis. Public databases and patient samples were used to investigate the aberrant expression of Oxoglutarate dehydrogenase-like (OGDHL) in ccRCC. The function of OGDHL in ccRCC growth and metastasis was evaluated through in vitro and in vivo functional experiments. Mechanistic insights were obtained through luciferase reporter assays, chromatin immunoprecipitation, RNA methylation immunoprecipitation, and mutagenesis studies. OGDHL mRNA and protein levels were significantly downregulated in ccRCC tissues. Upregulation of OGDHL expression effectively inhibited ccRCC growth and metastasis both in vitro and in vivo. Furthermore, FTO-mediated OGDHL m6A demethylation suppressed its expression in ccRCC. Mechanistically, low levels of OGDHL promoted TFAP2A expression by inhibiting ubiquitination levels, which then bound to the FASN promoter region and transcriptionally activated FASN expression, thereby promoting lipid accumulation and ERK pathway activation. Our findings demonstrate the impact of OGDHL on ccRCC progression and highlight the role of the FTO/OGDHL/TFAP2A/FASN axis in regulating ccRCC lipid metabolism and progression, providing new targets for ccRCC therapy.

## Introduction

Renal cell cancer (RCC) is a prevalent tumor in the urinary system. In 2021, RCC incidence in the United States accounted for 4.01% of all cancers and 46.39% of urinary system cancers [1]. Clear cell renal cell carcinoma (ccRCC), the most prevalent histological subtype of RCC, accounts for 85% of RCC cases [2]. Despite substantial innovations in ccRCC drug therapy, challenges remain concerning individual treatment responses and the development of drug resistance in patients [3, 4].

Metabolic reprogramming is one of the primary features of cancer, which refers to the adaptive changes that cancer cells make to meet nutrient supply under abnormal growth conditions [5]. CcRCC is characterized by a high degree of metabolic reprogramming, with dysregulation of lipid metabolism being one of the most striking changes [6]. The histological appearance of clear cytoplasm in ccRCC cells is due to lipid accumulation [7]. Endogenous lipid synthesis or exogenous lipid uptake is essential for the survival and proliferation of ccRCC cells [8]. The presence of large amounts of neutral lipids such as cholesteryl ester (CE) and triglyceride (TG) in ccRCC tumors suggests that lipids play an important role in ccRCC [9, 10]. In addition, increased expression of fatty acid (FA) synthesis genes, such as acetyl-CoA carboxylase (ACC) and fatty acid synthase (FASN), was also shown in the ccRCC-related gene expression profile [11].

Oxoglutarate dehydrogenase complex (OGDHC), a key rate-limiting enzyme in the tricarboxylic acid (TCA) cycle, converts α-ketoglutarate (αKG) to succinyl-CoA and generates ATP and NADH through oxidative phosphorylation. OGDHC binds to electron transport chain (ETC) complex I on the matrix side of the inner membrane and localizes in the mitochondrial matrix [12, 13]. Oxoglutarate dehydrogenase-like (OGDHL) is the major rate-limiting subunit of OGDHC [14]. The OGDHL downregulation inhibits the key mitochondrial multienzyme OGDHC activity, leading to αKG accumulation, reversing the oxidative decarboxylation of isocitrate dehydrogenase (IDH) in the TCA cycle, and leading to citrate accumulation and lipid synthesis [15]. The role of OGDHL in liver, colorectal, thyroid, breast, and pancreatic cancers has been investigated in prior work [16–20]. However, it has not been evaluated in ccRCC.

The m6A modification, the most common internal chemical modification of mRNA in mammalian cells [21], is widespread in transcripts and plays a crucial role in regulating mRNA maturation, translation, and degradation [22–24]. Methyltransferases (METTL3, METTL14, and WTAP) [25–27], demethylases (FTO and ALKBH5) [28, 29], and methylation reading proteins (YTH domain family proteins) [23, 30, 31] are primarily responsible for the m6A modification. The m6A regulates multiple physiological processes, including self-renewal, proliferation, migration, and invasion, by affecting the target gene expression [32]. Fat mass and obesity-associated protein (FTO) is an RNA m6A demethylase initially identified as a gene associated with obesity and energy metabolism due to its ability to affect lipogenesis [28, 33]. FTO is overexpressed in various human cancers to stimulate cancer cell metabolism, thereby inducing tumorigenesis and chemoresistance [34]. The FTO-m6A axis may serve as a new potential therapeutic target for human cancers. However, the mRNA regulatory mechanism and downstream targets of FTO in ccRCC remain unknown.

In this study, our aim was to elucidate the clinical significance and regulatory mechanisms of OGDHL in ccRCC. We found that OGDHL acts as a tumor suppressor by inhibiting the proliferation, migration, and invasion of ccRCC cells. Mechanistic studies revealed that FTO inhibits OGDHL expression in ccRCC through RNA m6A methylation, which downregulates FASN, leading to lipid accumulation and ERK pathway activation. We propose that the FTO/OGDHL/FASN/ERK signaling axis enriches our current understanding of ccRCC progression and metabolic reprogramming and may serve as a novel therapeutic target for ccRCC.

## Materials and methods

### Human ccRCC tissues, cell lines, and cell culture

The normal and ccRCC tissues were obtained from Wuhan Union Hospital and were approved by the Huazhong University of Science and Technology Committee. All patients received no antitumor therapy before surgery, and the postoperative pathological results were ccRCC. The HK-2, A498, 786-O, CAKI, and OSRC cell lines were purchased from American Type Culture Collection (ATCC, USA). The authenticity of cell lines was confirmed through STR (Short Tandem Repeat) profiling analysis, and their mycoplasma contamination status was assessed prior to experiments, ensuring the reliability of the results. The cells were cultured in DMEM (Gibco, USA) containing 1% penicillin-streptomycin and 10% fetal bovine serum (BI, USA) according to supplier recommendations. The cells were grown at 37 °C in a cell incubator with 5% CO_2_.

### RNA extraction and RT-qPCR

Tissue and cell RNA were treated with TRIzol reagent and extracted using an RNA extraction kit (BIOABSE, China). A 1 µg RNA was reverse transcribed using the Hifair® II 1st Strand cDNA Synthesis Kit (Yeasen, China). Real-time qPCR was performed in a StepOnePlus™ PCR system (Applied Biosystems) using SYBR Green qPCR Master Mix (MCE, USA). The GAPDH was used as an internal control for normalization. All primers used in the study are included in Supplementary File 1.

### RNA-sequencing analysis

Cellular RNA extraction and whole transcriptome sequencing were performed by Majorbio (China).

### Immunohistochemistry

The tissue blocks were embedded in paraffin and sliced. Tissue sections were dewaxed, rehydrated, and incubated with antibodies. Then, the tissue blocks were embedded in paraffin and sliced. Tissue sections were dewaxed, rehydrated, and antigen-retrieved in sequence. After 1 h of blocking with TBST containing 5% goat serum, the primary antibody was incubated overnight at 4 °C, and the secondary antibody was incubated the next day. The DAB and hematoxylin were used to observe immune complexes and nuclei, respectively (Biosharp, BS915, Hefei, China).

### Establishment of cell lines

Overexpressing lentiviruses for OGDHL (NM_018245), FASN (NM_004104), and control lentiviruses were purchased from GeneChem Corporation (Shanghai, China). The vector backbone for overexpressing lentivirus is “Ubi-MCS-3FLAG-SV40-EGFP-IRES-puromycin”. A498 cells and CAKI cells were infected to establish stable lines according to the manufacturer’s instructions. Puromycin (2 μg/mL, Sigma, USA) was used in established stable cell lines for further cell selection.

The OGDHL siRNA, FASN siRNA, and FTO siRNA were purchased from Genepharma Corporation (Shanghai, China). FTO and TFAP2A overexpression plasmids were purchased from GeneChem Corporation (Shanghai, China). Cell lines were transfected according to the manufacturer’s instructions, and cell experiments were completed within 24–120 h.

### Western blot analysis and antibodies

The cells were lysed with RIPA buffer (Beyotime, China) containing protease inhibitors (MCE, USA) and phenylmethanesulfonyl fluoride (PMSF) (Sigma, USA). Protein concentrations were determined using a BCA protein assay kit (Beyotime, China). Proteins were separated using 10% or 12% SDS-PAGE, transferred to PVDF membranes (Roche, Switzerland), and blocked with 5% skim milk (BD, USA) for 1 h. After washing, the samples were incubated with the primary antibody overnight at 4 °C. The next day, the PVDF membrane was incubated with the secondary antibody for 2 h at room temperature, and the protein level was detected using the ChemiDoc XRS + imaging system (Biorad, USA). The primary antibody was used at a 1:1000 dilution. Antibody product numbers are as follows: anti-OGDHL (A15475, ABclonal, China), anti-FASN (A19050, ABclonal, China), anti-TFAP2A (13019-3-AP, Proteintech, China), anti-FTO (A3861, ABclonal, China), anti-RUNX1 (A0400, ABclonal, China), anti-ARNT (A0972, ABclonal, China), anti-CyclinD1(A2708, ABclonal, China), anti-MMP2 (A19080, ABclonal, China), anti-MMP9 (A0289, ABclonal, China), anti-pERK (AP0485, ABclonal, China), anti-ERK (A4782, ABclonal, China), anti-GAPDH (A19056, ABclonal, China). The original blots of the western blotting can be found in the Original data files.

### Cell Counting Kit 8 (CCK8) cell proliferation assay

In 96-well plates, 2 × 10^3^/well cells were seeded. The cells were treated at 0, 24, 48, 72, and 96 h using CCK8 (YEASEN, China) according to the instructions. The absorbance of treated cells was measured at 450 nm using a NanoDrop 2000 spectrophotometer (NanoDrop Technologies, USA).

### Colony formation assays

Approximately 10^3^ cells were seeded and cultured in a 6-well plate for 2 weeks. The cells were fixed with methanol, stained with 0.05% crystal violet (Servicebio, G1014, Wuhan, China), and colonies were observed.

### Transwell assays

Cells pre-cultured in serum-free medium were placed in transwell chambers (Corning Costar Corp, USA) with or without Matrigel (BD Company, USA) plating for migration and invasion experiments. After 24 h of culture, cells were fixed with methanol and stained with 0.05% crystal violet.

### RNA m6A dot blot assay

Poly (A) RNA was spotted on the nylon membrane (GE, USA). Then Membranes were UV cross-linked and blocked for 15 min before incubation with m6A antibody (ABE572, 1:1000, Merck Millipore) overnight at 4 °C. The secondary antibody was incubated for 1 h at room temperature. The cleaned nylon membrane was soaked with ECL reagent (Thermo, USA) for 1 min and visualized using ChemiDoc XRS + imaging system.

### Oil red staining

Saturated oil red was diluted to 2/5 of the original concentration with ultrapure water and heated at 60 °C in a water bath for 30 min to promote fusion. After fixing cells with 4% paraformaldehyde, oil red dye was added and stained for 1 h at room temperature.

### Triglyceride detection

The cells were resuspended in the 10 cm dish, and the supernatant was removed. The pellet was dissolved with 500 µL of 2% TritonX-100 for 40 min. The TG content was determined using a Triglyceride assay kit (Nanjing Jianchen, China).

### Chromatin immunoprecipitation (Chip) assay

SimpleChIP® Kit (CST, USA) was used to perform a ChIP assay according to the manufacturer’s protocol to verify the regulatory mechanism between TFAP2A and FASN. The fragmented chromatin from lysed 10^7^ A498 cells was immunoprecipitated with protein G agarose beads and anti-H3 (positive control), anti-TFAP2A, or anti-immunoglobulin G (negative control), respectively, followed by chromatin elution. The primer sequences in Supplementary File 1 are used for PCR analysis.

### Methylated RNA immunoprecipitation (MeRIP) assay

The OGDHL m6A immunoprecipitation of OGDHL was performed according to the standard protocol of the Magna Methylated RNA Immunoprecipitation (MeRIP) m6A Kit (Merck Millipore, USA). Quantitative analysis was performed using RT-qPCR.

### Luciferase reporter assays

Cells were transfected with specific plasmids using Lipofectamine 2000 in 12-well plates. Luciferase activity was determined according to the standard protocol of dual luciferase detection reagent (Promega, USA). pRL-T was an internal control (Promega, USA). The truncated plasmid of the promoter region of OGDHL and the mutated and wild-type plasmids of the 3′UTR region of OGDHL were purchased from Genechem. The construction vectors were GV272.

### Tumor formation and metastasis assays

The ability of tumors to form in vivo was evaluated using a subcutaneous metastasis model. We assigned a random number to each nude mouse and randomized their allocation into experimental groups based on the order of these random numbers. Approximately 2 × 10^6^ CAKI cells infected with vector, OGDHL, OGDHL + vector, OGDHL + FASN lentivirus were prepared in 100 µL cell suspension and injected into the armpits of 6-week-old BALB/c nude mice (Vital River, China). Six mice were used in each group for the subcutaneous tumor model constructed using the vector OGDHL. For the functional recovery model, each group of subcutaneous tumor mice consisted of five individuals. Tumor-seeded nude mice were euthanized 49 days later. Tumor volume and mass were recorded. During this period, tumor volume was recorded weekly with a vernier caliper. After the fixation and embedding of subcutaneous tumors, IHC staining was performed.

In vivo, tumor metastasis ability was evaluated using a tail vein metastasis model. A 100 µL of cell suspension containing 1 × 10^6^ A498 cells stably infected with the above lentivirus was injected into the tail vein of nude mice, with each metastasis model consisting of four mice per group. After 49 days, a part of the nude mice was anesthetized by intraperitoneal injection of 10% chloral hydrate. Fluorescence distribution and intensity were observed using a small animal in vivo fluorescence imager (Lago X, USA) to analyze tumor metastasis. The other mice were euthanized and dissected. Liver tissue was fixed and embedded, sliced, and stained with H&E.

For this animal experiment, blinding was implemented to minimize potential biases during data collection and analysis. The investigator conducting the experiment and assessing the outcomes was blinded to the group assignments.

### Bioinformatics analysis

Gene mRNA data and patient clinical parameters in the bioinformatics analysis were obtained from The Cancer Genome Atlas (TCGA) (https://portal.gdc.cancer.gov) Kidney Clear Cell Carcinoma (KIRC) database. SPSS Statistics 22.0 was used to analyze gene expression, patient survival, and related clinical parameters. GraphPad Prism 7.0 software was used to generate curves and histograms. The gene sets related to metabolism and lipid metabolism that were used to screen genes were obtained from the Oncomine database (https://www.oncomine.org). Enrichment pathways for ccRCC RNAseq data in the TCGA-KIRC database were identified using GSEA v4.1.0 for Windows (UC San Diego, USA).

### Statistical analysis

Graphpad prism 7.0 was used to analyze mean, SD, and SEM and to plot receiver operating characteristic (ROC) and area under the curve (AUC). Statistical analysis was performed using SPSS Statistics 2.0 for *t*-test or analysis of variance. For *t*-tests, a minimum sample size of *n* ≥ 3 was considered. Pearson analysis was used to analyze the correlation between genes. Statistical significance was considered for *p* < 0.05.

## Results

### OGDHL is significantly downregulated in ccRCC and associated with poor patient outcomes

**Fig. 1:**
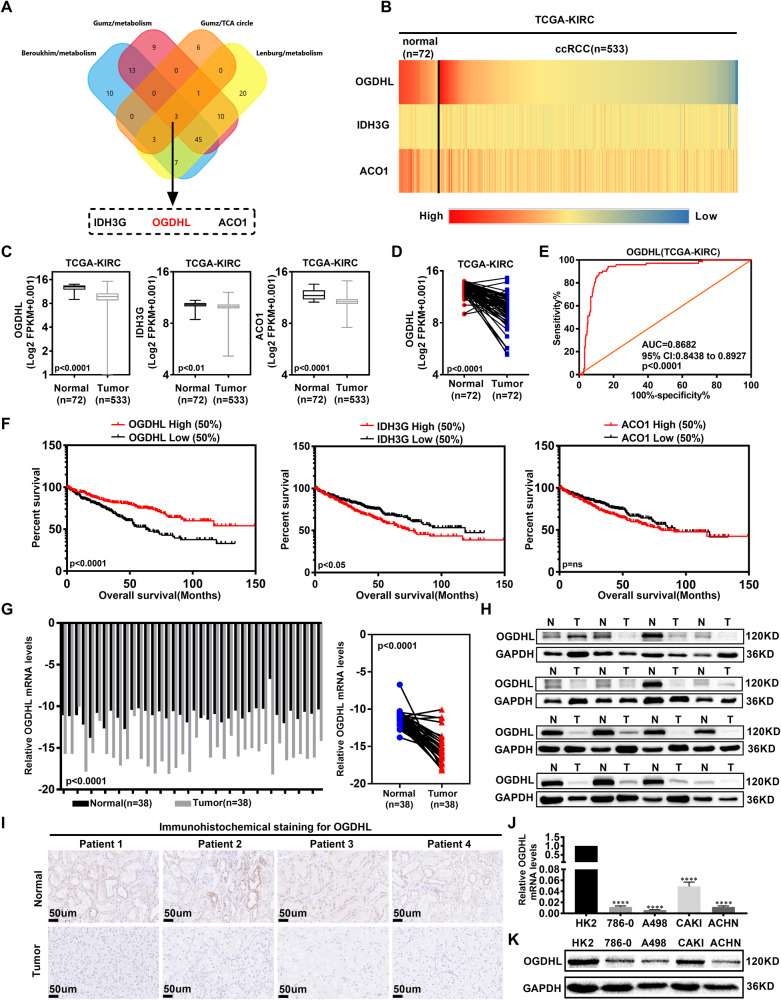
OGDHL is significantly downregulated in ccRCC and associated with poor patient outcomes. **A** A Venn diagram consists of three independent sets of metabolism-related genes and TCA cycle genes from the Oncomine database (https://www.oncomine.org). **B** Heatmap of mRNA expression levels of OGDHL, IDH3G, and ACO1 in 533 ccRCC tissues and 72 paracancerous tissues in the TCGA database. **C** Boxplots of OGDHL, IDH3G, ACO1 mRNA expression in 533 ccRCC tissues and 72 paracancerous tissues in TCGA database. Independent samples *t*-test, *p* < 0.05, was considered statistically significant. **D** The mRNA expression of OGDHL in 72 ccRCC tissues and their paired adjacent normal tissues in the TCGA database. Paired samples *t*-test, *p* < 0.05, was considered statistically significant. **E** ROC curves were drawn according to the expression levels of OGDHL in tumor samples and normal samples in the TCGA-KIRC database to evaluate the sensitivity and specificity of its diagnostic ability. ROC curve for OGDHL (AUC = 0.8682 95% CI: 0.8438–0.8927; *p* < 0.0001). **F** Kaplan–Meier survival curves of OGDHL, IDH3G, and ACO1 based on the TCGA database. *p*-Values were obtained by Log-rank (Mantel–Cox) test. **G** Statistical plot of mRNA expression of OGDHL in 38 pairs of ccRCC tissues and adjacent non-malignant tissues. Paired samples *t*-test, *p* < 0.05, was considered statistically significant. **H** Protein levels of OGDHL in clinically obtained ccRCC tissues and adjacent non-malignant tissues (*n* = 16). **I** Immunohistochemical sections of 4 pairs of ccRCC tissues and adjacent non-malignant tissues incubated with OGDHL primary antibody. Scale bar: 50 μm. **J** OGDHL mRNA expression levels in 4 ccRCC cell lines (786-0, A498, CAKI, ACHN) and control cell line (HK2). *t*-test, ****p < 0.0001, ***p < 0.001, **p < 0.01, and *p < 0.05 (independent-samples *t*-test for statistics). **K** OGDHL protein levels in 4 ccRCC cell lines (786-0, A498, CAKI, ACHN) and control cell line (HK2).

Based on the metabolic abnormalities characteristic of ccRCC, we employed the Oncomine database to screen for ccRCC-related metabolic genes. OGDHL, IDH3G, and ACO1 were identified as potential genes influencing lipid metabolism in ccRCC (Fig. 1A). Their expression and prognostic value in ccRCC were assessed using the TCGA database. In comparison to adjacent normal tissues, the mRNA expression levels of OGDHL, IDH3G, and ACO1 were downregulated in ccRCC (Fig. 1B–D, fig. S1A). ROC curve analysis demonstrated that OGDHL exhibited a significantly higher AUC than IDH3G and ACO1, indicating its superior diagnostic value (Fig. 1E, Fig. S1B). Subsequently, Kaplan–Meier survival analysis revealed that OGDHL expression could better predict the survival time of ccRCC patients compared to IDH3G and ACO1 (Fig. 1F). The diagnostic and prognostic values of the above genes in ccRCC led to identifying OGDHL as a target gene for follow-up studies by comparing the expression levels. Furthermore, bioinformatics analysis was performed on the relevant clinical parameters and OGDHL mRNA expression of 532 groups of ccRCC patients in the TCGA-KIRC database. Table 1 shows that OGDHL downregulation correlated with the progression of Tumor, Node, Metastasis (TNM) staging, and G grades in ccRCC (Fig. S1C). Subsequently, subgroup survival analysis of ccRCC clinical parameters showed that OGDHL downregulation reduced the overall survival and disease-free survival time of ccRCC patients with different TNM stages and G grades (Figs. S2 and S3). Finally, univariate and multivariate Cox regression analysis identified OGDHL as an independent risk factor for overall survival and disease-free survival in ccRCC patients (Table 2). Moreover, we demonstrated the low OGDHL expression in ccRCC through a series of experiments. The detection of OGDHL mRNA and protein levels in tumor tissue and paracancerous samples from clinical ccRCC patients showed that its expression was significantly reduced in ccRCC (Fig. 1G–I). Consistent with the above results, the OGDHL mRNA and protein expression levels were significantly downregulated in ccRCC cell lines compared to control cell lines (Fig. 1J, K). In summary, OGDHL is significantly downregulated in ccRCC and is highly associated with the prognosis of ccRCC patients.

**Table 1:** Correlation between OGDHL mRNA expression and clinicopathological parameters of ccRCC patients.

Parameter		Total (cases, [%])	OGDHL mRNA expression (cases, [%])		*P* value
			Low (*n* = 266)	High (*n* = 266)	
Age (years)					–
	≦60	266 (50)	133	133	
	>60	266 (50)	133	133	
Gender					0.000^*^
	Male	344 (64.7)	204	140	
	Female	188 (35.3)	62	126	
T stage					0.002^*^
	T1 + T2	342 (64.3)	153	189	
	T3 + T4	190 (35.7)	113	77	
N stage					–
	N0 + NX	516 (97.0)	257	259	
	N1	16 (3.0)	9	7	
M stage					0.000^*^
	M0 + MX	451 (85.1)	209	242	
	M1	79 (14.9)	55	24	
G grades					0.013^*^
	G1 + G2	242 (45.7)	108	134	
	G3 + G4	287 (54.3)	157	130	
TNM stage					0.000^*^
	I + II	324 (61.2)	139	185	
	III + IV	205 (38.8)	126	79	

“*” indicates that the *p*-value is statistically significant, *p* < 0.05.

Relevant clinical data of ccRCC patients are all from the TCGA-KIRC database.

**Table 2:** Univariate and multivariate analyses of OGDHL mRNA level and patient overall survival or disease-free survival.

Variable	Overall survival				Disease-free survival			
	Univariate analysis		Multivariate analysis^c^		Univariate analysis		Multivariate analysis^c^	
	HR^a^ (95% CI^b^)	*P* value	HR^a^ (95% CI^b^)	*P* value	HR^a^ (95% CI^b^)	*P* value	HR^a^ (95% CI^b^)	*P* value
Age (≤60 years vs. >60 years)	1.778 (1.310–2.414)	<0.001^*^	1.614 (1.186–2.197)	0.002^*^	1.433 (0.913–2.248)	0.117	–	–
Gender (female vs. male)	1.055 (0.775–1.435)	0.735	–	–	1.199 (0.7351–1.956)	0.463	–	–
T stage (T1 or T2 vs. T3 or T4)	3.154 (2.331–4.267)	<0.001^*^	–	–	6.766 (4.089–11.195)	<0.001^*^	–	–
N stage (N0 or NX vs. N1)	3.852 (2.086–7.115)	<0.001^*^	2.334 (1.246–4.370)	0.008^*^	6.989 (3.323–14.696)	<0.001^*^	2.918 (1.377–6.187)	0.005^*^
M stage (M0 or MX vs. M1)	4.376 (3.213–5.958)	<0.001^*^	–	–	12.237 (7.753–19.315)	<0.001^*^	4.661 (2.801–7.756)	<0.001^*^
G grade (G1 or G2 vs. G3 or G4)	2.564 (1.826–3.600)	<0.001^*^	1.753 (1.229–2.498)	0.002^*^	5.373 (2.957–9.763)	<0.001^*^	2.991 (1.617–5.532)	<0.001^*^
TNM stage (Stage I + II vs. Stage III + IV)	3.840 (2.800–5.265)	<0.001^*^	2.959 (2.116–4.140)	<0.001^*^	11.429 (6.285–20.786)	<0.001^*^	4.281 (2.159–8.488)	<0.001^*^

^a^Hazard ratio, estimated from Cox proportional hazard regression model.

^b^Confidence interval of the estimated HR.

^c^Multivariate models were adjusted for T, N, M classification, age, and gender.

“*” indicates that the *p*-value is statistically significant, *p* < 0.05.

Relevant clinical data of ccRCC patients are all from the TCGA-KIRC database.

### FTO-mediated m6A modification of OGDHL mRNA in ccRCC

**Fig. 2:**
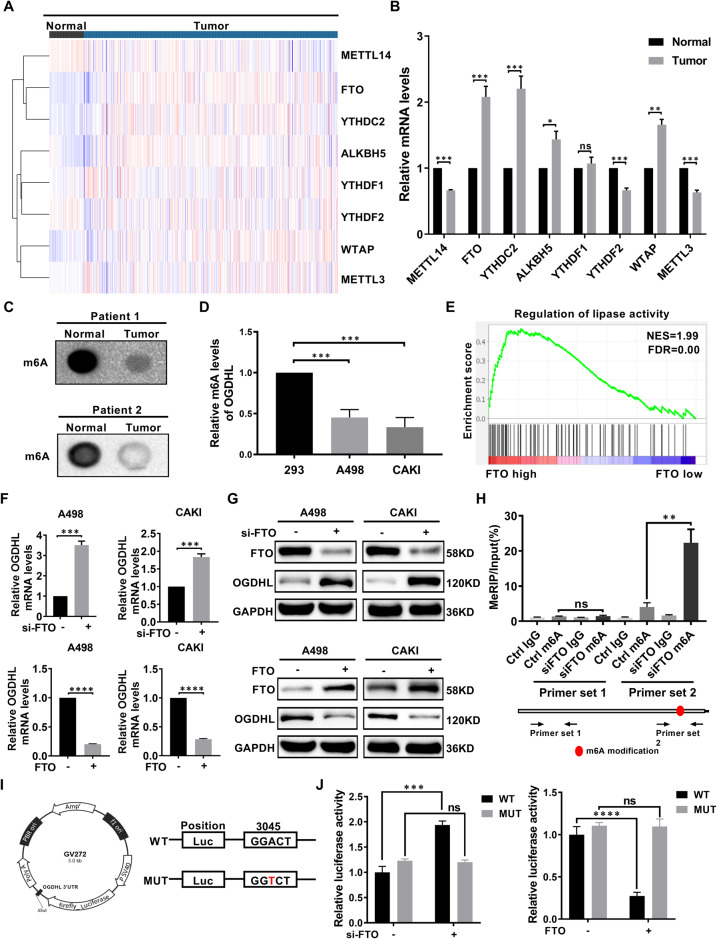
FTO-mediated m6A modification of OGDHL mRNA in ccRCC. **A** Heatmap of differential gene expression in ccRCC tissues and normal tissues. **B** mRNA levels of m6A-regulated enzymes in ccRCC and paracancerous normal tissues (Tissues are derived from clinical, surgical samples). **C** Overall mRNA m6A levels in ccRCC and normal tissue samples were determined by RNA m6A dot blot. **D** Overall mRNA m6A levels of ccRCC cell line versus control 293 cell line. **E** A GSEA correlation chart based on the TCGA-KIRC database with FTO expression as the premise for screening. FDR < 25% and *p* < 0.05 was considered statistically significant. **F** qRT-PCR was used to verify the mRNA levels of FTO knockdown or overexpressed. **G** Western blot analysis was used to verify the protein levels of FTO knockdown or overexpressed. **H** Knockdown of FTO promoted m6A methylation in OGDHL mRNA by m6A MeRIP analysis. **I** Wild-type OGDHL 3′UTR and OGDHL 3′UTR with mutations at the m6A consensus sequence were cloned into a luciferase reporter gene. Mutations in the m6A modified region were generated by substituting thymine for adenosine. **J** Relative luciferase activity of wild-type and mutant OGDHL 3′UTR reporter vectors in FTO knockdown or overexpressing A498 cells.

The above studies have demonstrated that the OGDHL mRNA expression level is downregulated in ccRCC, but the mechanism leading to abnormal OGDHL expression remains unclear. m6A modification is the most prevalent internal mRNA modification in mammalian cells, which plays a role in the malignant progression of tumors by regulating the expression of relevant genes [35]. There is a significant difference in the expression of m6A modification-related genes between paired and unpaired ccRCC samples in the TCGA database. (Fig. 2A, Fig. S4A). The mRNA levels of m6A-modified core genes exhibited differential expression between tumor and non-tumor tissues of ccRCC patients (Fig. 2B). Furthermore, the RNA dot-blot assay demonstrated a significant decrease in global m6A abundance in ccRCC (Fig. 2C). The same results were obtained when detecting m6A modification levels in ccRCC and control cell lines (Fig. 2D). Subsequently, correlation analysis of OGDHL mRNA revealed a significant negative association with the m6A demethylase FTO and a significant positive association with the m6A methyltransferase WTAP (Fig. S4B). GSEA results of FTO showed that FTO regulates lipid metabolism in ccRCC (Fig. 2E). Building upon the correlation at the mRNA level and functional relevance observed between FTO and OGDHL in ccRCC, FTO was selected as a potential regulatory gene for OGDHL and pursued further investigations. FTO knockdown or overexpression ccRCC cell lines were constructed by transfecting siRNA and overexpression plasmids to clarify the FTO regulation on OGDHL (Fig. S4C, D). Silencing the FTO expression increased OGDHL expression and vice versa (Fig. 2F, G). Knockdown of FTO significantly increased m6A levels of OGDHL mRNA (Fig. 2H). A potential m6A site was identified in the 3′ UTR region near the OGDHL termination codon using the m6A modification site prediction server SRAMP (http://www.cuilab.cn/sramp). To eliminate m6A modification, adenines within the consensus m6A sequence were replaced by thymidines to generate the mutant OGDHL 3’ UTR reporter vector (Fig. 2I). In FTO knockout cells, the wild-type OGDHL 3′ UTR showed a significant increase in luciferase activity compared to the control cells, while the mutant OGDHL 3′ UTR did not exhibit such changes. Similar results were observed in cells overexpressing FTO, indicating that FTO-regulated m6A modification modulates the expression of OGDHL at this modified site (Fig. 2J).

### OGDHL inhibits the progress of ccRCC in vitro

**Fig. 3:**
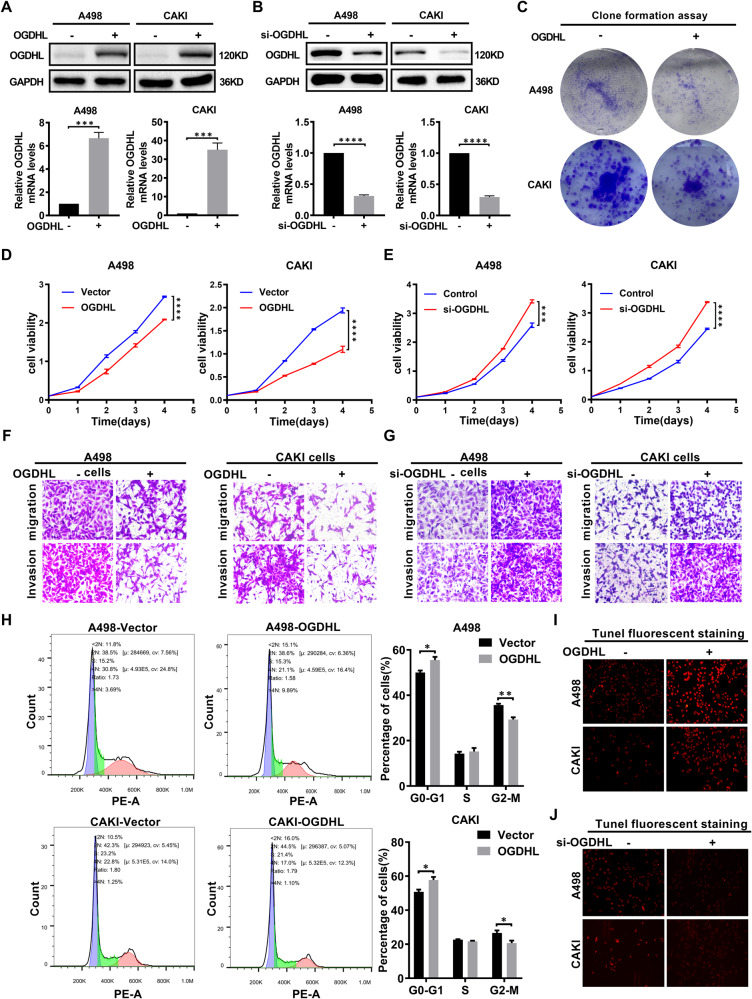
OGDHL inhibits the progress of ccRCC in vitro. The results are expressed as the mean ± SEM of three independent experiments, and there are at least three replicates in each independent experiment. *t*-test, ****p < 0.0001, ***p < 0.001, **p < 0.01, and *p < 0.05 (independent-samples *t*-test for statistics). **A** Western blotting and qPCR were used to verify the overexpression of OGDHL at the protein and mRNA levels, respectively. **B** Western blotting and qPCR confirmed the knockdown of OGDHL at the protein and mRNA levels, respectively. **C** Colony formation experiments of ccRCC cell lines infected with OGDHL overexpressing and control lentivirus. **D** The CCK8 assay was used to determine the level of proliferation of OGDHL overexpressing and control ccRCC cell lines. **E** The CCK8 assay was used to determine the level of proliferation of knockdown OGDHL and control ccRCC cell lines. **F** Transwell assays were used to determine the migration and invasion abilities of OGDHL-overexpressing and control ccRCC cell lines. **G** Transwell assays were used to determine the migration and invasion abilities of knockdown OGDHL and control ccRCC cell lines. **H** Flow cytometric analysis was performed on OGDHL overexpressing and control ccRCC cell lines. Representative images and quantification of results are presented. **I** Tunel fluorescence staining was used to reflect the apoptosis level of OGDHL overexpression and control ccRCC cell lines. **J** Tunel fluorescence staining was used to reflect the apoptosis level of OGDHL knockdown and control ccRCC cell lines.

We discovered that m6A modification-mediated OGDHL was significantly downregulated in ccRCC, suggesting patient prognosis. OGDHL stable overexpressed or knocked down ccRCC cell lines (A498 and CAKI) were established to study further the function of OGDHL in ccRCC (Fig. 3A, B). The OGDHL overexpression significantly inhibited ccRCC cell proliferation, whereas OGDHL knockdown increased cell proliferation (Fig. 3C–E). Consistently, the detection of cell migration and invasion abilities showed that overexpression of OGDHL significantly inhibited the migration and invasion abilities of ccRCC cells (Fig. 3F, Fig. S5A), while knockdown of OGDHL promoted these abilities (Fig. 3G, Fig. S5B). Flow cytometry analysis of cell cycle distribution revealed that OGDHL overexpression increased the proportion of cells in the G0–G1 phase while decreasing the proportion of cells in the G2-M phase (Fig. 3H), whereas OGDHL knockdown had the opposite result (Fig. S5C, D). Fluorescent staining for apoptosis indicated that OGDHL overexpression increased the apoptosis in ccRCC cells (Fig. 3I, Fig. S5E), whereas knockdown of OGDHL had the opposite trend (Fig. 3J, Fig. S5F). Our data established that OGDHL plays an important role in controlling ccRCC cell growth, colony formation, cell cycle, and cell death.

### OGDHL mitigates lipid accumulation in ccRCC

**Fig. 4:**
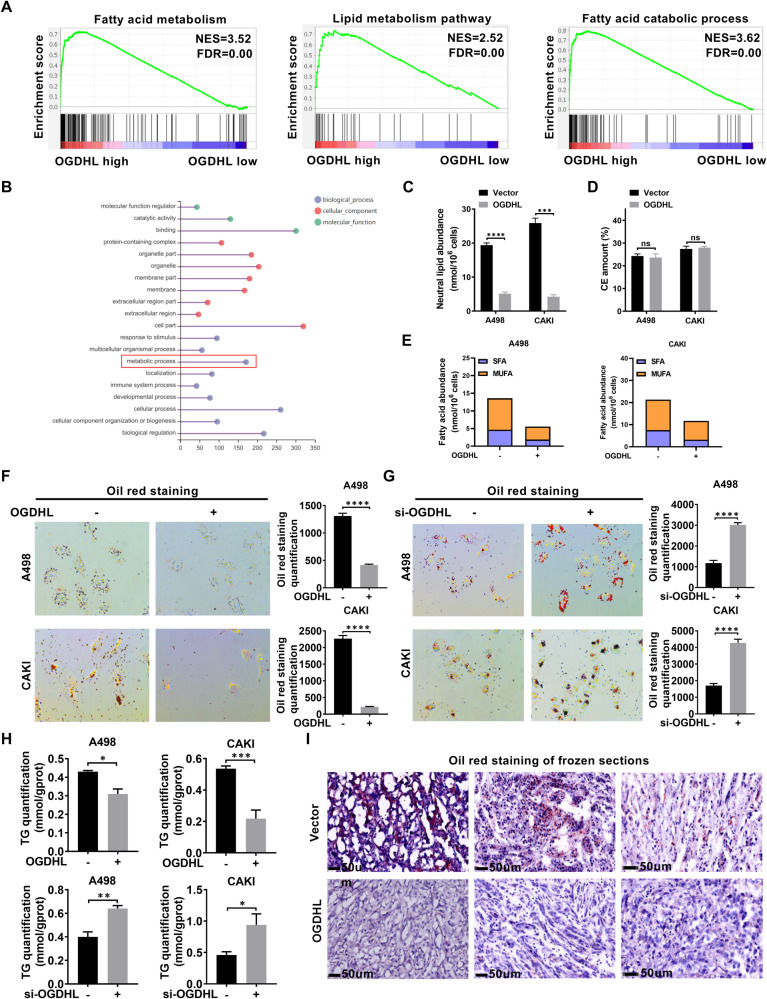
OGDHL mitigates lipid accumulation in ccRCC. **A** The GSEA correlation charts are based on the TCGA-KIRC database with OGDHL expression as the premise for screening. FDR < 25% and *p* < 0.05 was considered statistically significant. **B** GO pathway analysis of whole transcriptome sequencing. **C**–**E** The abundance of neutral lipids (**C**), cholesterol esters (**D**), and saturated fatty acids (SFA), and monounsaturated fatty acids (MUFA) in neutral lipids fractions were analyzed in ccRCC cells overexpressing OGDHL. **F** Oil red O staining micrographs and quantitative analysis of OGDHL-overexpressing and control ccRCC cell lines. **G** Oil red O staining micrographs and quantitative analysis of knockdown OGDHL and control ccRCC cell lines. **H** Relative TG (mmol/gprot) levels in OGDHL overexpressing or knockdown ccRCC cell lines. **I** Oil red cryosection staining of subcutaneous tumor tissue. Nude mice subcutaneously transplanted tumor models were established by inoculating OGDHL stably overexpressing or control A498 cells.

The accumulation of intracellular lipid droplets is a key contributing factor to the aberrant lipid metabolism in ccRCC progression [36]. In this study, OGDHL was identified as a potential gene involved in regulating ccRCC metabolism, and its specific role in metabolic regulation was further elucidated through enrichment analyses. GSEA results demonstrated significant enrichment of OGDHL in various lipid metabolic pathways, including fatty acid metabolism, lipid metabolic pathways, and fatty acid biosynthesis (Fig. 4A, Fig. S6A). GO and KEGG analyses based on the transcriptome sequencing data of A498 cells overexpressing OGDHL further revealed a pronounced concentration of lipid metabolism (Fig. 4B, Fig. S6B). The lipidomic analysis confirmed that neutral lipids were decreased in ccRCC cells with upregulated OGDHL expression (Fig. 4C), primarily manifested as decreases in saturated and monounsaturated fatty acids rather than cholesteryl esters (CE) (Fig. 4D, E). Oil red staining results showed that lipid accumulation was significantly reduced in ccRCC cell lines overexpressing OGDHL, whereas it significantly increased in OGDHL-knockdown cell lines (Fig. 4F, G). The quantification results of triglycerides indicated that OGDHL overexpression reduced the intracellular triglyceride content in ccRCC cells, while knockdown had the opposite effect (Fig. 4H). Finally, Oil Red staining of xenograft tumors demonstrated that OGDHL effectively inhibits lipid accumulation in ccRCC in vivo (Fig. 4I). The above research results indicate that OGDHL is a regulatory factor of lipid accumulation in ccRCC both in vitro and in vivo.

### OGDHL inhibits ccRCC progression by inhibiting FASN-regulated lipid metabolism and ERK signaling pathways

**Fig. 5:**
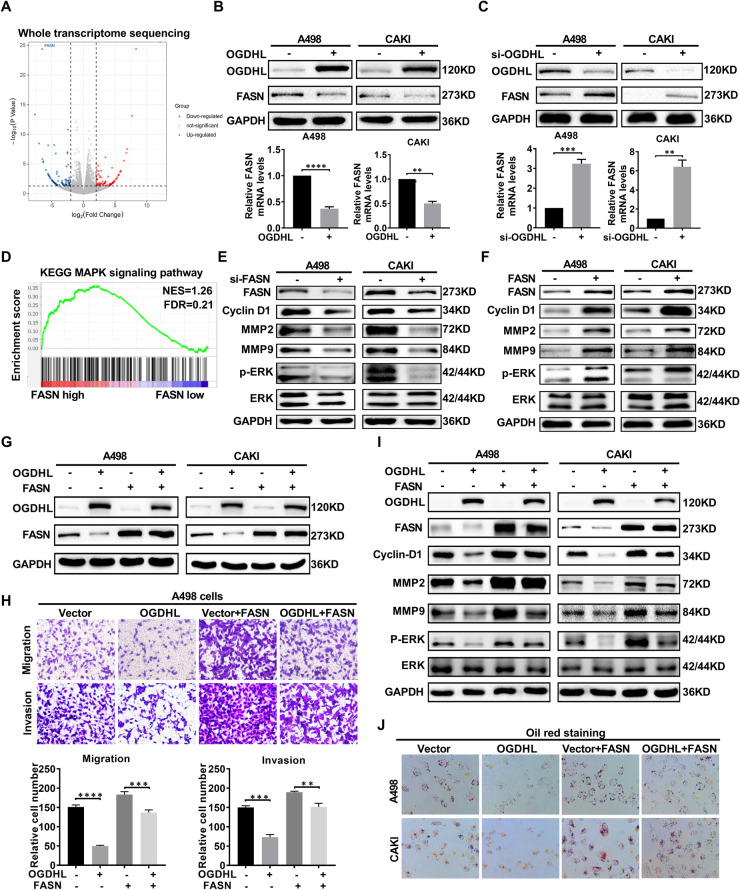
OGDHL inhibits ccRCC progression by inhibiting FASN-regulated lipid metabolism and ERK signaling pathways. **A** Volcano plot of whole transcriptome sequencing data after OGDHL overexpression. FASN was a significantly downregulated gene after OGDHL overexpression. **B**. Protein and mRNA levels of FASN after stable overexpression of OGDHL were detected by western blot analysis and qRT-PCR; *t*-test, ****p < 0.0001, ***p < 0.001, **p < 0.01, *p < 0.05 (independent samples *t*-test). **C** Protein and mRNA levels of FASN after OGDHL knockdown were detected by western blot analysis and qRT-PCR; *t*-test, ****p < 0.0001, ***p < 0.001, **p < 0.01, *p < 0.05 (independent samples *t*-test). **D** The GSEA correlation charts are based on the TCGA-KIRC database with FASN expression as the premise for screening. FDR < 25% and *p* < 0.05 was considered statistically significant. **E** Expression of key members of the MAPK signaling pathway was detected in FASN knockdown and control ccRCC cell lines by western blotting. **F** Expression of key members of the MAPK signaling pathway was detected in FASN-overexpressing and control ccRCC cell lines by western blotting. **G** Western blot analysis was used to detect the protein levels of OGDHL and FASN in the established stable cell lines. **H** Transwell assay results for migration and invasion of stable cell lines; *t*-test, ****p < 0.0001, ***p < 0.001, **p < 0.01, and *p < 0.05 (independent-samples *t*-test for statistics). **I** Western blotting detected the expression of key members of MAPK signaling pathway in ccRCC functionally restored cell lines constructed by overexpressing OGDHL and overexpressing FASN lentivirus. **J** Oil red O staining photograph of ccRCC cells.

It is well-known that OGDHL functions through the tricarboxylic acid cycle, and we have demonstrated that OGDHL reduces lipid accumulation in ccRCC, which is clearly another feature of OGDHL in tumor progression. To further elucidate the specific target of OGDHL in regulating the lipid metabolism level of ccRCC, we screened for the gene FASN through transcriptome sequencing and Oncomine database analysis of 147 genes directly related to lipid metabolism (Fig. 5A, Fig. S6C). FASN, the only human lipogenic enzyme capable of de novo fatty acid synthesis, catalyzes the endogenous synthesis of fatty acids. Overexpression of OGDHL leads to a decrease in both FASN protein and mRNA levels (Fig. 5B), while knockdown of OGDHL shows the opposite effect (Fig. 5C). The enrichment results from GSEA confirmed that FASN regulates ccRCC lipid metabolism (fig. S6D, E). Simultaneously, we found that FASN significantly affected MAPK signaling in ccRCC (Fig. 5D). Therefore, FASN knockdown and overexpression ccRCC cell lines were established to investigate the alterations in protein levels of ERK pathway genes (Fig. S6F). As shown in Fig. 5E, F, FASN knockdown significantly decreased ERK phosphorylation, whereas overexpression of FASN increased ERK phosphorylation. The FASN was screened on the premise of OGDHL overexpression and lipid metabolism and played an important role in regulating the ERK pathway. Thus, we hypothesized that FASN plays a key role in the OGDHL’s tumor suppressor effect. We used FASN-overexpressing lentivirus to construct a functional recovery model in ccRCC cells stably overexpressing OGDHL to test the above hypothesis (Fig. 5G). As shown in Fig. S6G, the upregulation of FASN could clearly reverse the inhibition of cell proliferation caused by OGDHL overexpression. Simultaneously, the detection of cell migration and invasion ability can also get consistent results (Fig. 5H, Fig. S6H). Western blot analysis of ERK pathway genes in the above stable lines indicated that FASN upregulation could reverse the increased phosphorylation of ERK caused by OGDHL overexpression (Fig. 5I). Subsequently, cellular lipid accumulation was identified using intracellular oil red staining and quantification. Consistently, FASN upregulation reversed the reduction in lipid accumulation caused by overexpression of OGDHL (Fig. 5J, Fig. S6I). We can conclude that FASN downregulation inhibits lipid accumulation and the ERK pathway, a key link in the inhibition of ccRCC progression by OGDHL.

### OGDHL controlled FASN by TFAP2A transcriptional regulation

**Fig. 6:**
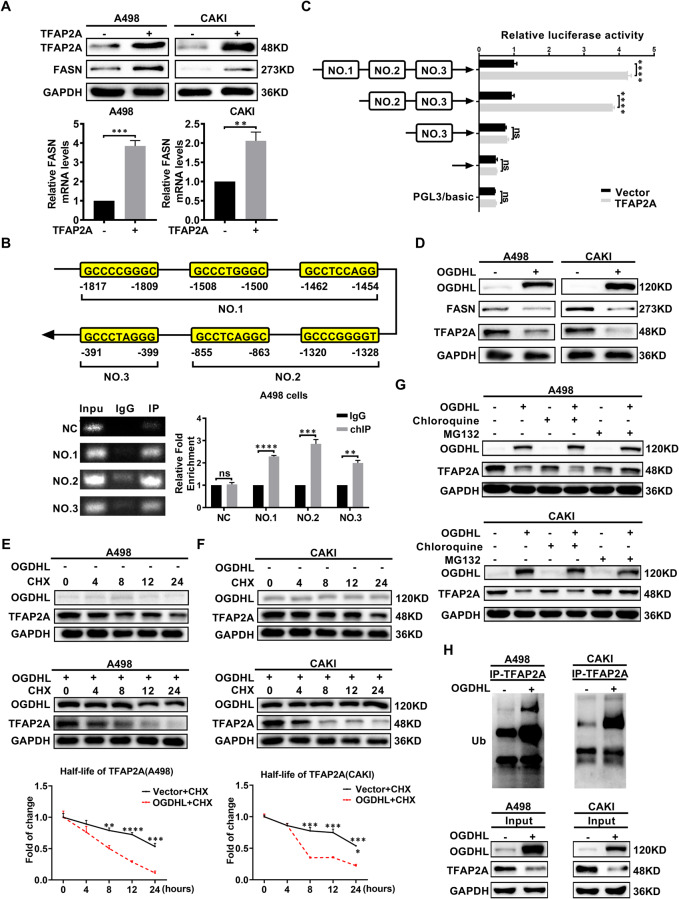
OGDHL controlled FASN by TFAP2A transcriptional regulation. The results are expressed as the mean ± SEM of three independent experiments, and there are at least three replicates in each independent experiment. *t*-test, ****p < 0.0001, ***p < 0.001, **p < 0.01, and *p < 0.05 (independent-samples *t*-test for statistics). **A** FASN protein and mRNA levels after TFAP2A overexpression were detected by western blotting and qRT-PCR. **B** Primers were constructed segmentally according to the predicted binding site of TFAP2A on the FASN promoter region. Agarose gel electrophoresis and qRT-PCR were used to visualize the results of ChIP experiments. **C** Fluorescence detection of A498 cells transfected with truncated plasmids showed that TFAP2A binds to FASN promoter NO. 2 (−1382 ~ −855) or NO. 3 (−399 ~ −391). **D** Western blot analysis was used to detect the protein levels of FASN and TFAP2A in OGDHL-overexpressing ccRCC cell lines. **E**, **F** Protein stability assay in A498 cells overexpressing OGDHL. Cells were treated with 50 μM cycloheximide (CHX), harvested at specific time points (0 h, 4 h, 8 h, 12 h, 24 h), and the protein levels of OGDHL and TFAP2A were detected by western blot analysis. **G** A498 and CAKI cells were treated independently with 20 µM MG132 and chloroquine for 12 h, and the expression of TFAP2A protein was analyzed by western blot analysis. **H** Negative controls and OGDHL lentivirus-infected ccRCC cell lines were immunoprecipitated with TFAP2A antibody and incubated with ubiquitin (Ub) antibody for western blot analysis.

In the above studies, FASN was an important downstream of OGDHL in reducing tumor lipid accumulation and tumor suppressor. We conducted a series of mechanistic studies to determine how OGDHL regulates FASN. Since FASN is a gene screened based on the results of whole transcriptome sequencing after OGDHL overexpression, related literature also shows that FASN can be transcriptionally regulated [37–39]. We speculate that OGDHL can regulate FASN transcriptionally by affecting the expression level of a certain transcription factor. TFAP2A, RUNX1, and ARNT were identified as potential transcription factors for FASN by searching the JASPAR program database (Fig. S7A). Scatterplot analysis based on the TCGA-KIRC database showed that the mRNA levels of TFAP2A and FASN were positively correlated in ccRCC patients, with the highest correlation (Fig. S7B). Furthermore, Western blot analysis revealed that OGDHL overexpression led to a downregulation of the TFAP2A protein level while having no impact on the protein levels of RUNX1 and ARNT (Fig. S7C). Therefore, we identified TFAP2A as an intermediate gene that regulates FASN expression levels by OGDHL and carried out a specific mechanism study. TFAP2A-overexpressing ccRCC cell lines were constructed by transfection of overexpression plasmid. FASN mRNA and protein levels were upregulated in TFAP2A-overexpressing ccRCC cell lines (Fig. 6A). We constructed three binding regions using the predicted binding sites of TFAP2A in the FASN promoter region (Fig. S7A). Chromatin immunoprecipitation (ChIP) analysis indicated that TFAP2A is directly bound to NO. 1, NO. 2, and NO. 3 regions of the FASN gene promoter region in A498 cells (Fig. 6B). The dual luciferase reporter gene assay was performed on A498 cells overexpressing TFAP2A. After removing the NO. 2 or NO. 3 regions, the increase in luciferase activity mediated by TFAP2A overexpression was significantly reversed, while the removal of NO.1 had no noticeable effect. It is suggested that the region of NO. 2 or NO. 3 is the potential site of TFAP2A transcriptional activation of FASN (Fig. 6C). The TFAP2A transcriptionally regulated FASN, and it is necessary to understand further the specific mechanism by which OGDHL acts on TFAP2A. As shown in Fig. 6D and Fig. S7D, OGDHL upregulation decreased levels of TFAP2A and FASN proteins, whereas downregulation of OGDHL resulted in increased levels of TFAP2A and FASN proteins. Subsequently, GSEA results indicated a correlation between OGDHL and protein degradation in ccRCC, suggesting a potential mechanism through which OGDHL regulates downstream protein levels (Fig. S7E). The OGDHL overexpression can significantly accelerate the degradation rate of TFAP2A in ccRCC cell lines (Fig. 6E, F). This indicated that OGDHL could significantly reduce the protein stability of TFAP2A. Protein degradation pathways included the lysosome-dependent pathway and the ubiquitin-proteasome pathway. We treated ccRCC cell lines stably overexpressing OGDHL with the lysosomal inhibitor chloroquine and the proteasome inhibitor MG132 to determine the specific pathway by which OGDHL affects TFAP2A degradation. The inhibitory effect of OGDHL on TFAP2A protein level was significantly reversed in the MG132-treated group, while there was almost no reverse effect in the chloroquine-treated group (Fig. 6G). The OGDHL mainly regulated the TFAP2A expression through the ubiquitin-proteasome pathway. Subsequent western blot analysis showed that the OGDHL upregulation increased the ubiquitination level of TFAP2A (Fig. 6H). The above mechanism experiments indicate that OGDHL negatively regulates the protein level of TFAP2A by promoting ubiquitination, thereby inhibiting the transcriptional activation of FASN by TFAP2A and reducing FASN expression.

### OGDHL inhibits ccRCC progression in vivo

**Fig. 7:**
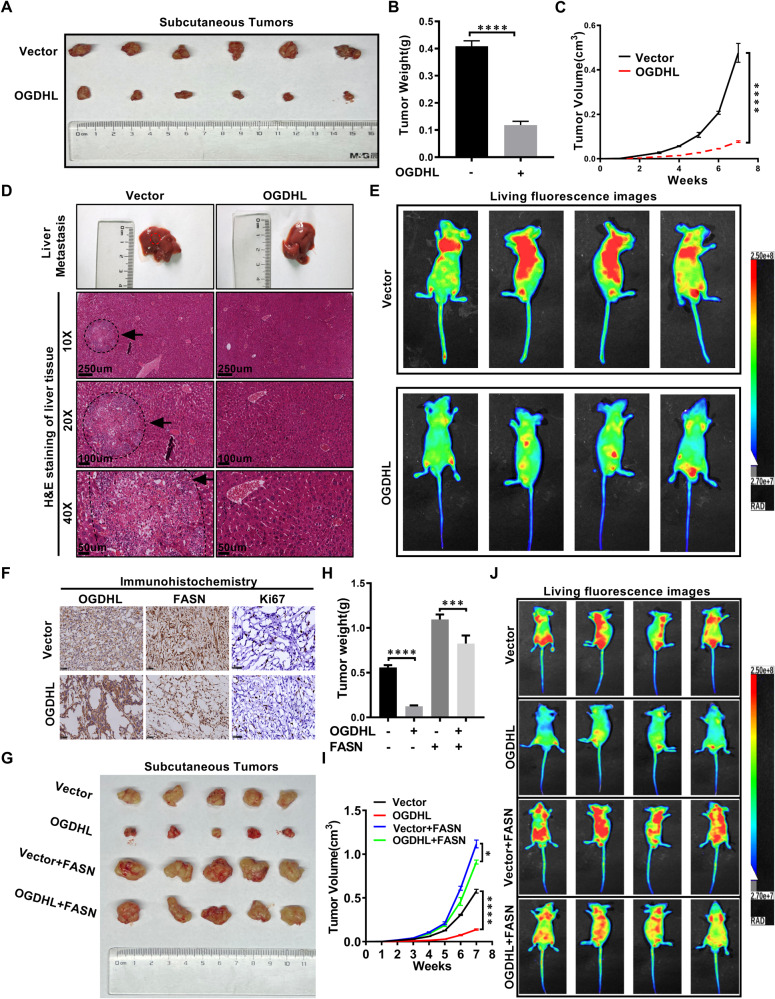
OGDHL inhibits ccRCC progression in vivo. **A**, **B** A498 cells infected with vector or OGDHL lentivirus were injected subcutaneously into nude mice, respectively. After the 7th week, the tumor size and body weight of nude mice in each group were measured. The data are expressed as the mean ± SEM of each group of tumors; *t*-test, ****p < 0.0001, ***p < 0.001, **p < 0.01, and *p < 0.05 (independent-samples *t*-test for statistics). **C** The tumor volume of each group was measured every week. This graph is drawn based on the relationship between the number of weeks after tumor cell implantation and tumor size (mm^3^). Data are expressed as the mean ± SEM from tumors of each group; *t*-test, ****p < 0.0001, ***p < 0.001, **p < 0.01, and *p < 0.05 (independent-samples *t*-test for statistics). **D** H&E staining of liver tissue of nude mice in OGDHL overexpression group and control group. Scale bar: 250 μm, 100 μm, 50 μm. **E** Living fluorescence images of OGDHL-overexpressing and control nude mice in a metastasis model. **F** Immunohistochemical (IHC) staining was used to detect the levels of OGDHL, FASN, and tumor malignancy (Ki67) in the subcutaneous xenograft model. Scale bar: 50 μm. **G** Nude mouse subcutaneous tumor model was established by injecting A498 cells infected with vector, OGDHL, vector + FASN, OGDHL + FASN lentivirus. **H** Subcutaneous tumor weight (g) of nude mice after 7 weeks. **I** Volume growth curve of subcutaneous tumor in nude mice within 7 weeks. **J** Living fluorescence images in the metastasis model.

The role of OGDHL in vivo has become the exploration focus due to cell experiments. Consistent with the in vitro results, the subcutaneous tumor xenograft model with high OGDHL expression grew more slowly than controls, and the tumor mass and volume were smaller (Fig. 7A–C). H&E staining and in vivo imaging of small animal liver based on nude mice tail vein metastasis demonstrated that OGDHL overexpression could significantly reduce the level of tumor metastasis (Fig. 7D, E). Next, the immunohistochemical results of subcutaneous xenografts also revealed that FASN expression level and tumor malignancy index KI67 decreased after OGDHL overexpression (Fig. 7F). Consistent with in vitro experiments, FASN upregulation can reverse the growth and metastasis inhibition caused by OGDHL overexpression (Fig. 7G–J). Overexpression of OGDHL inhibited ccRCC progression in vivo, and upregulation of FASN, a vital downstream gene of OGDHL, reversed this inhibition in vivo.

**Fig. 8:**
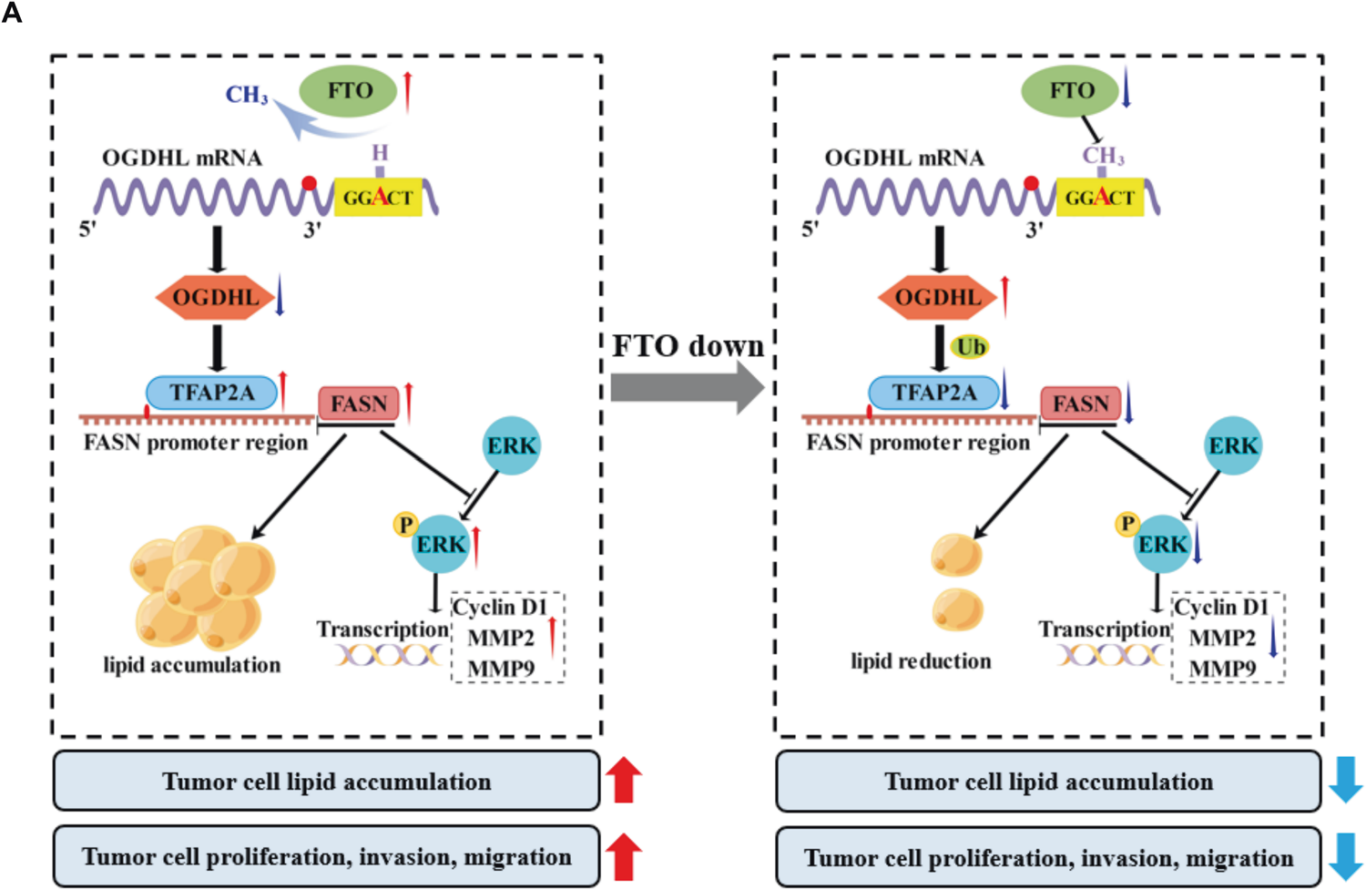
The specific mechanism of OGDHL-mediated regulation of lipid metabolism in ccRCC. **A** Working Model of FTO/OGDHL/TFAP2A/FASN axis in ccRCC.

In summary, we have established a model wherein FTO, highly expressed in ccRCC cells, mediates the m6A demethylation of OGDHL, resulting in its downregulation. The decreased OGDHL levels lead to an increase in TFAP2A expression by attenuating its ubiquitin-mediated degradation. The elevated TFAP2A, in turn, binds to the promoter region of FASN, activating its transcription and consequently fostering intracellular lipid accumulation, along with the activation of the ERK pathway. This entire cascade propels the proliferation, migration, and invasion of ccRCC cells. Upon FTO downregulation, the 3′UTR region of OGDHL mRNA undergoes m6A methylation, resulting in its upregulation, thereby exerting negative control over the entire pathway. Ultimately, this culminates in cellular lipid depletion, diminishing the proliferative, migratory, and invasive capabilities of ccRCC cells (Fig. 8A).

## Discussion

Metabolic alterations are one of the hallmarks of cancer, and metabolic reprogramming has been considered a common phenomenon in tumors [40, 41]. Lipid accumulation is a key feature of metabolic reprogramming in ccRCC, and studies have shown that it promotes tumor cell growth and invasion [42–44]. However, the direct evidence and mechanism of lipid metabolism affecting the biological behavior of ccRCC cells have not been elucidated. Our study identified OGDHL as a critical gene involved in the metabolic reprogramming of ccRCC and mainly regulates lipid metabolism processes. Low OGDHL expression in ccRCC was strongly associated with a poor prognosis. Furthermore, the mechanism study revealed that the m6A methylation modification of OGDHL mRNA by FTO inhibited the OGDHL expression, whereas low levels of OGDHL upregulated the FASN expression by inhibiting the ubiquitination level of TFAP2A. We discovered that the FTO/OGDHL/TFAP2A/FASN axis promotes lipid accumulation and activates the ERK pathway, thereby driving ccRCC progression.

The m6A modification is the most common among more than 100 chemical modifications of human mRNA that have been discovered [45]. The m6A modification dysregulation is closely related to the occurrence and development of cancer. FTO, the first demethylase identified in m6A modification, plays an important role in tumor progression and adipogenesis regulation [46, 47]. Our study identified that FTO mediates m6A modification of OGDHL mRNA and inhibits its expression in ccRCC. However, whether FTO affects the mRNA stability of OGDHL requires further investigation.

The OGDHL regulates mitochondrial function, cell cycle, apoptosis, and energy metabolism in tumor cells [16–18, 20]. According to the above studies, OGDHL, as a pleiotropic protein, inhibits the growth and metastasis of ccRCC in vitro and in vivo. Thus, we confirmed that OGDHL acts as a tumor suppressor in ccRCC. Simultaneously, we discovered that OGDHL could reverse lipid accumulation in ccRCC cells. The ccRCC has massive lipid accumulation because the lipid synthesis rate exceeds the decomposition rate [48, 49]. Related studies have confirmed that the FA accumulation in ccRCC is due to its enhanced synthesis, in which ACC and FASN play crucial roles as rate-limiting enzymes in the FA synthesis pathway [50–52]. Lipid accumulation can maintain tumor cell energy homeostasis to promote tumor progression [53], so lipid accumulation is currently considered a marker of tumor aggressiveness [54, 55].

Mitogen-activated protein kinases (MAPKs), also known as extracellular signal-regulated kinases (ERKs), function to transmit upstream signals to downstream effectors to regulate processes such as cell proliferation, differentiation, and apoptosis [56]. ERK1 and ERK2, members of the MAPK family, are frequently mutated in human cancers, and targeting the MAPK pathway has been considered an effective cancer treatment strategy [57–60]. Multiple studies have confirmed that ERK signaling is an important pathway for promoting ccRCC growth and metastasis [61–63]. ERK signaling promotes tumor cell proliferation, invasion, and migration by elevating cyclin D1 and matrix metalloproteinases (MMPs). Cyclin D1 is an important regulatory target of the G1-S phase transition and is closely related to tumor cell proliferation [64]. The MMPs enhance tumor cell motility by degrading the extracellular matrix (ECM) [65]. This study confirmed that the effect of OGDHL on the biological behavior of ccRCC is caused by the inactivation of ERK signaling, which inhibits the expression of cyclin D1, MMP2, and MMP9.

We predicted and screened the transcription factor TFAP2A that regulates FASN. TFAP2A is a transcription factor that belongs to the activator protein 2 (AP-2) family and can bind to specific GC-rich sequences to activate or repress downstream genes [66] and is critical for the regulation of gene expression during early development and oncogenic processes [67]. Our study determined that TFAP2A binds to the FASN promoter region to activate the FASN expression in ccRCC. Bioinformatics analysis and related reports showed that OGDHL could affect the protein stability of downstream genes [68, 69]. We demonstrated that OGDHL promotes the ubiquitination of TFAP2A to downregulate its protein levels. Overall, low levels of OGDHL upregulated the FASN expression by inhibiting the ubiquitination of TFAP2A, implying that the low OGDHL levels in ccRCC could promote cellular lipid accumulation by upregulating lipid synthesis through increased FASN levels.

## Conclusion

This study reveals that the abnormal downregulation of OGDHL in ccRCC is regulated by FTO-mediated m6A modification. Low levels of OGDHL negatively regulate the transcription of FASN via TFAP2A, causing activation of the ERK pathway and increased lipid synthesis in ccRCC. Therefore, this study uncovers important regulatory mechanisms for the ERK pathway and lipid metabolism in ccRCC and provides an opportunity for the development of ccRCC treatment strategies targeting FASN or OGDHL m6A modification.

## Supplementary information

Supplementary File 1

Supplementary File 2

Original Data File

## Data Availability

The data that support the findings of this study are available from the corresponding author upon reasonable request. The sequencing datasets has been deposited in one of the recommended data repositories (Science Data Bank, http://www.scidb.cn/). The private link of sequencing datasets is https://www.scidb.cn/s/neuiqm.
