# Supplementary figures and images for: The m6A modification-mediated OGDHL exerts a tumor suppressor role in ccRCC by downregulating FASN to inhibit lipid synthesis and ERK signaling
Source: Cell Death Dis. 2023 Aug 25;14(8):560. doi: 10.1038/s41419-023-06090-7 (PMC10457380; doi:10.1038/s41419-023-06090-7)

**Supplementary File 1**


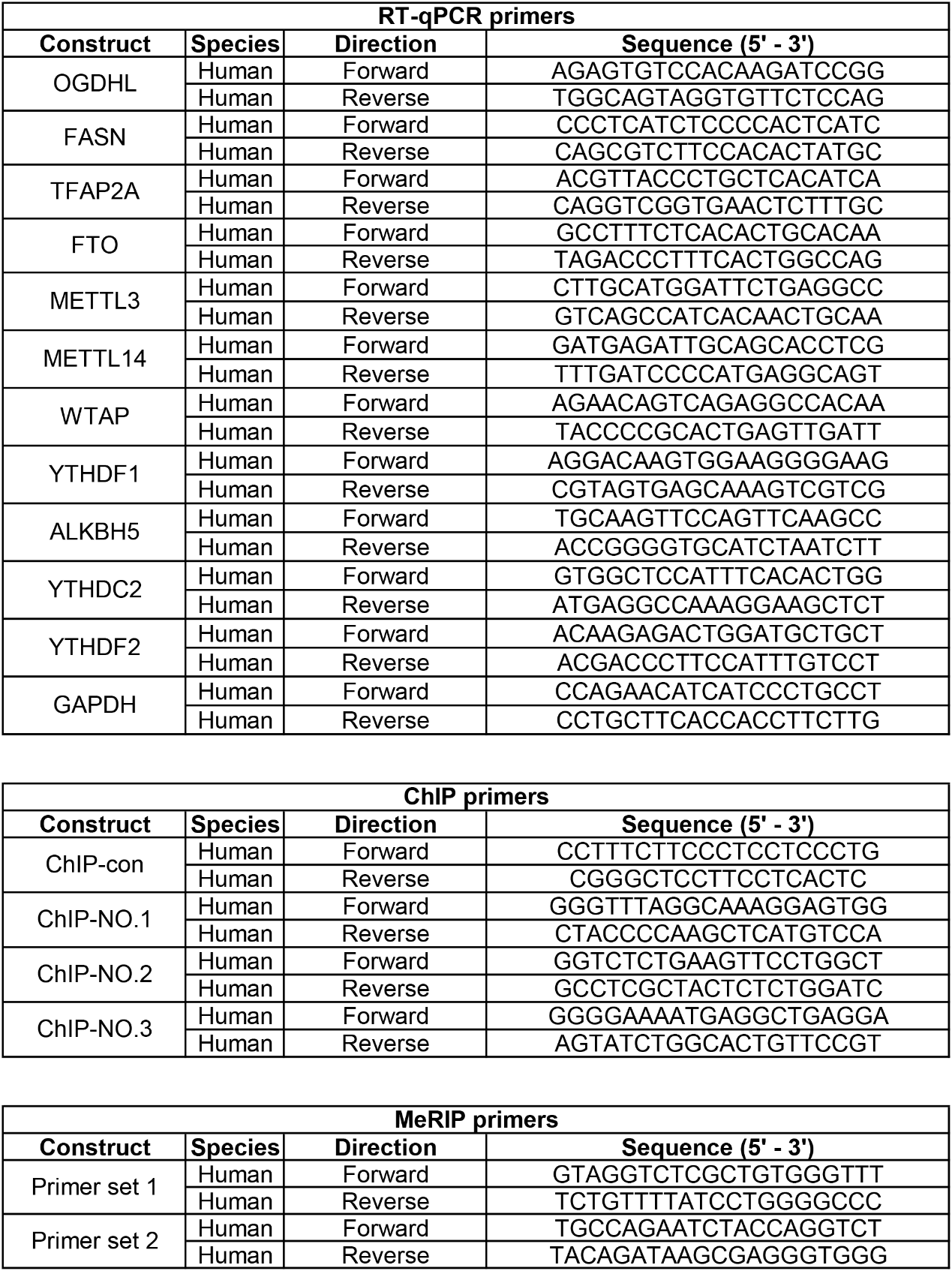

Supplement: Supplementary file 1 [file 41419_2023_6090_MOESM1_ESM.docx]

**Original blots of the western blotting**


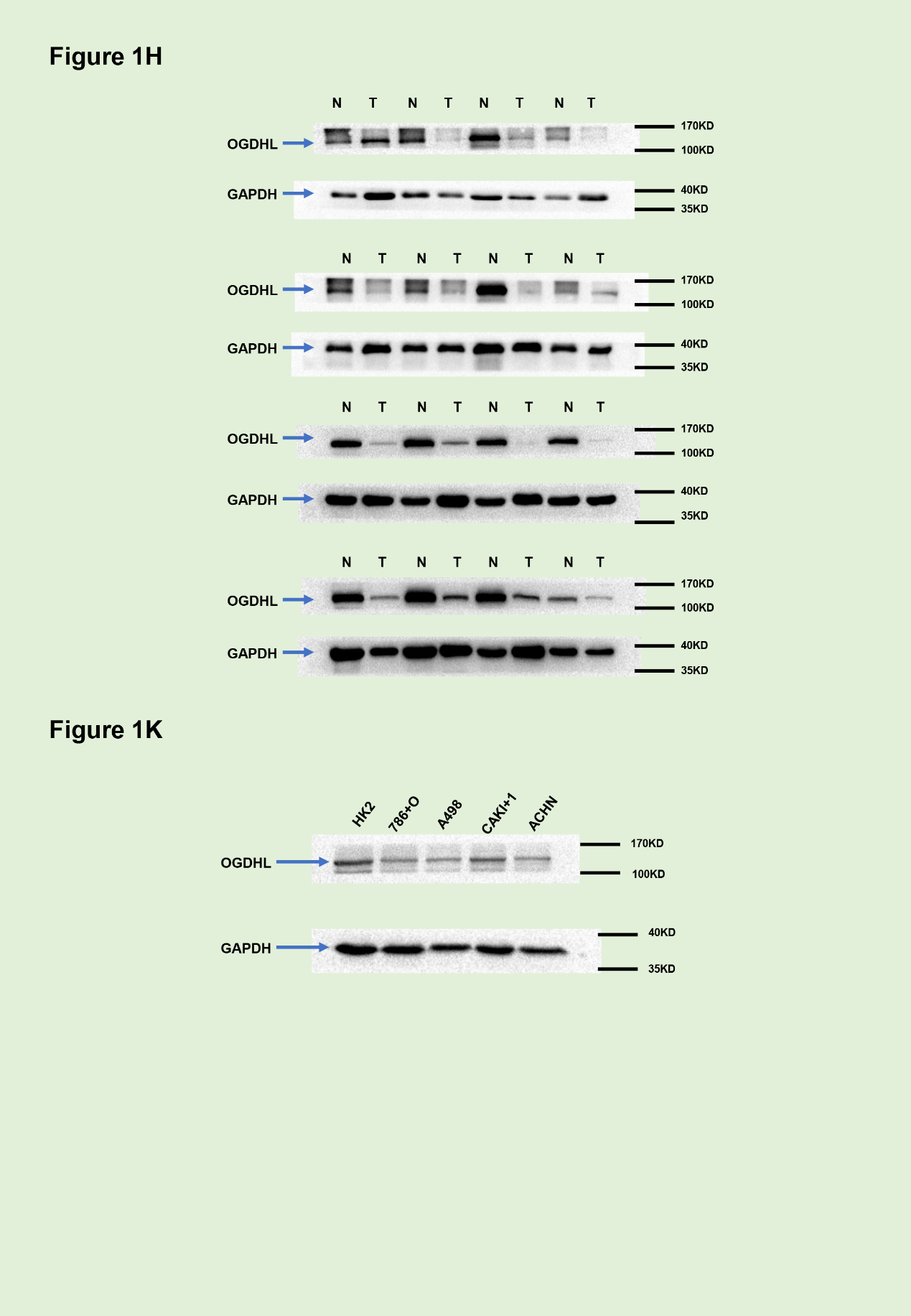

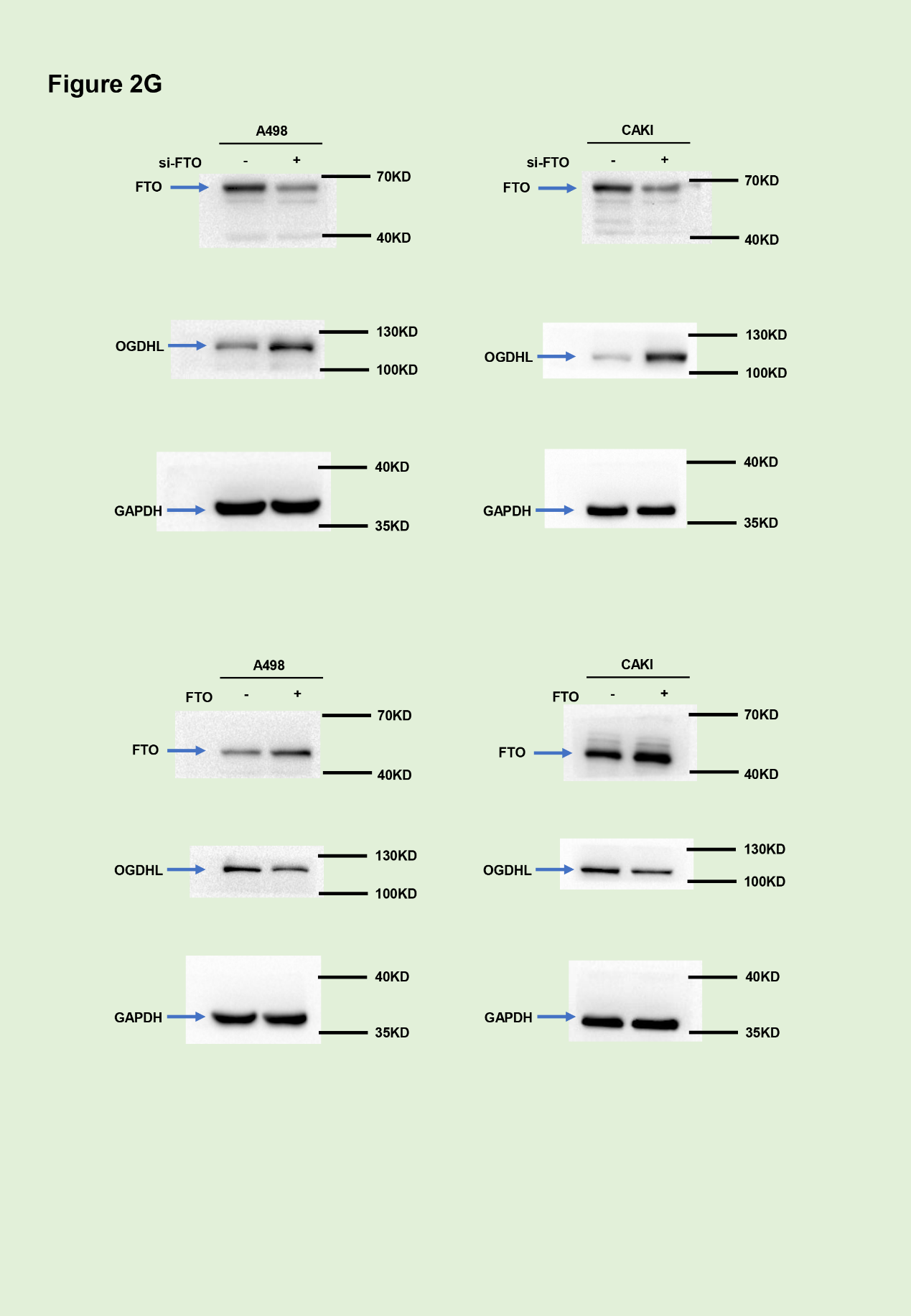

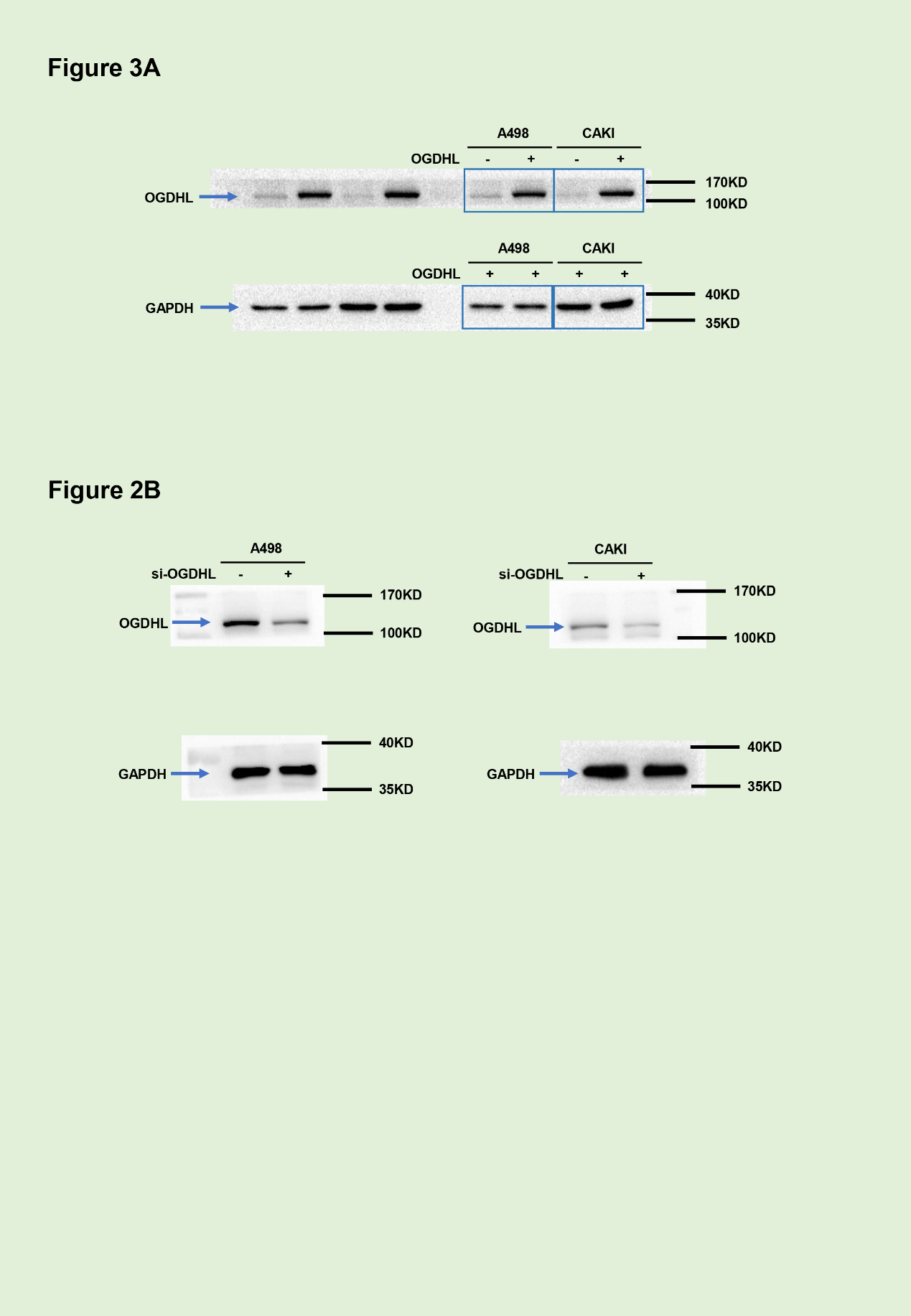

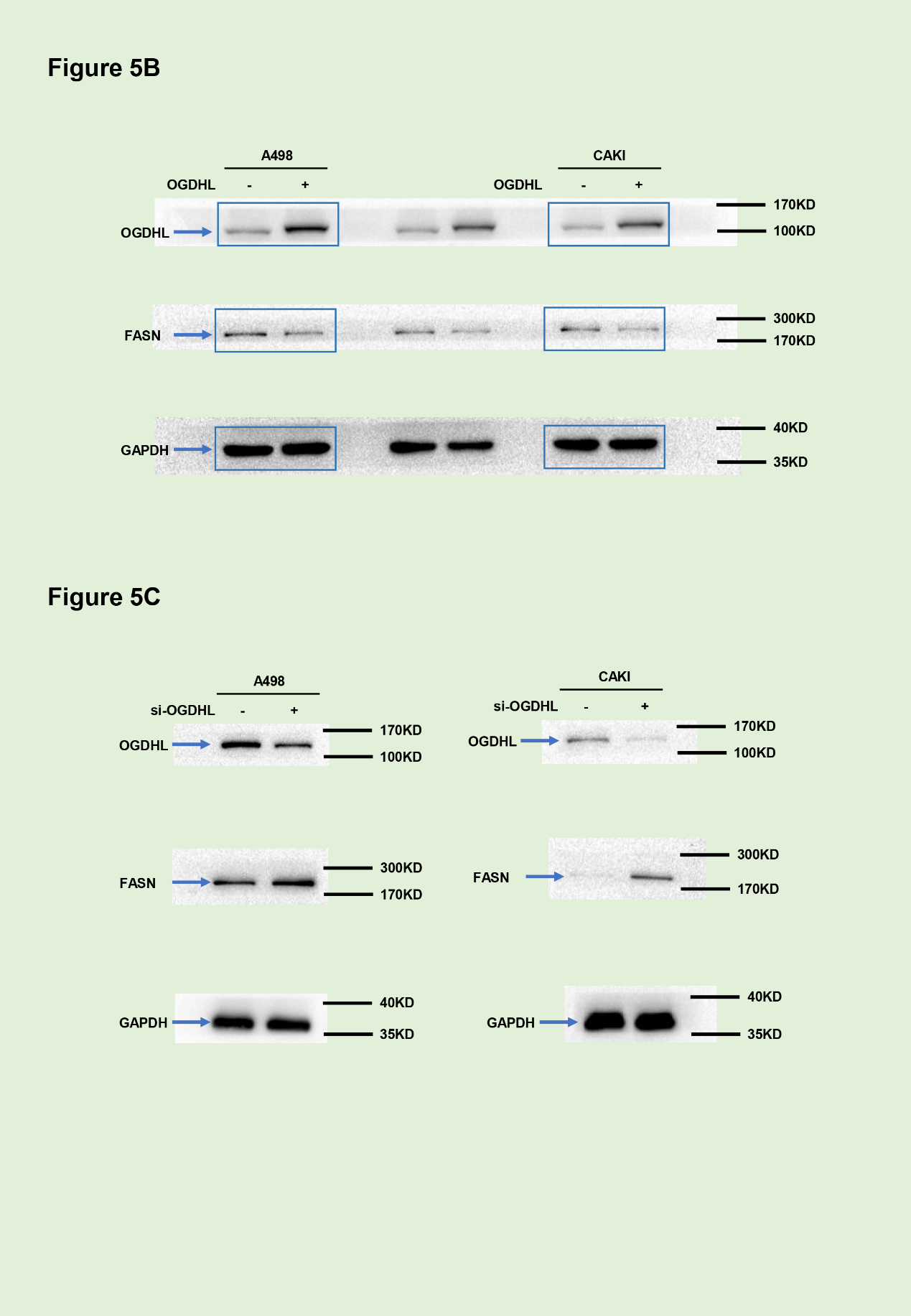

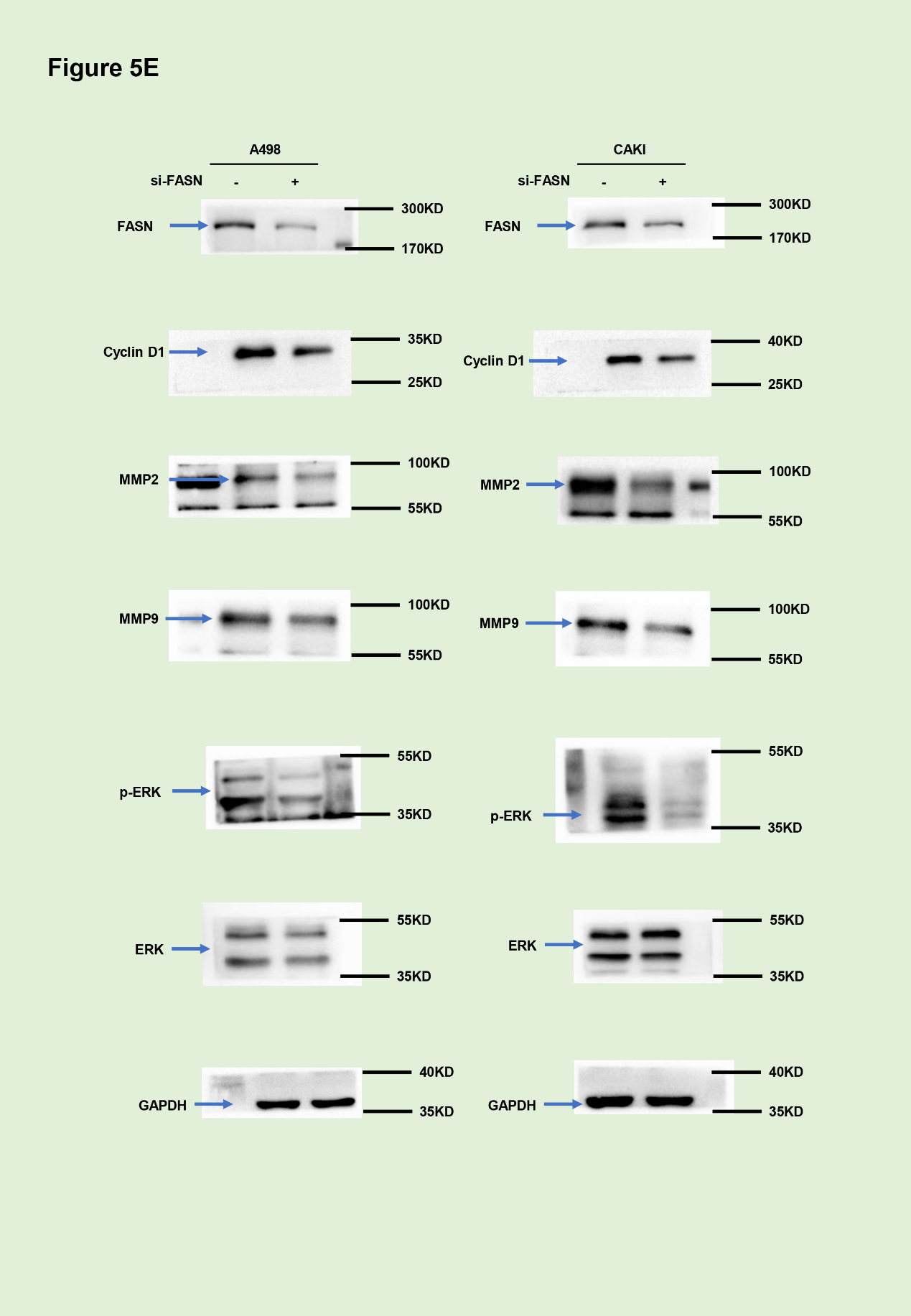

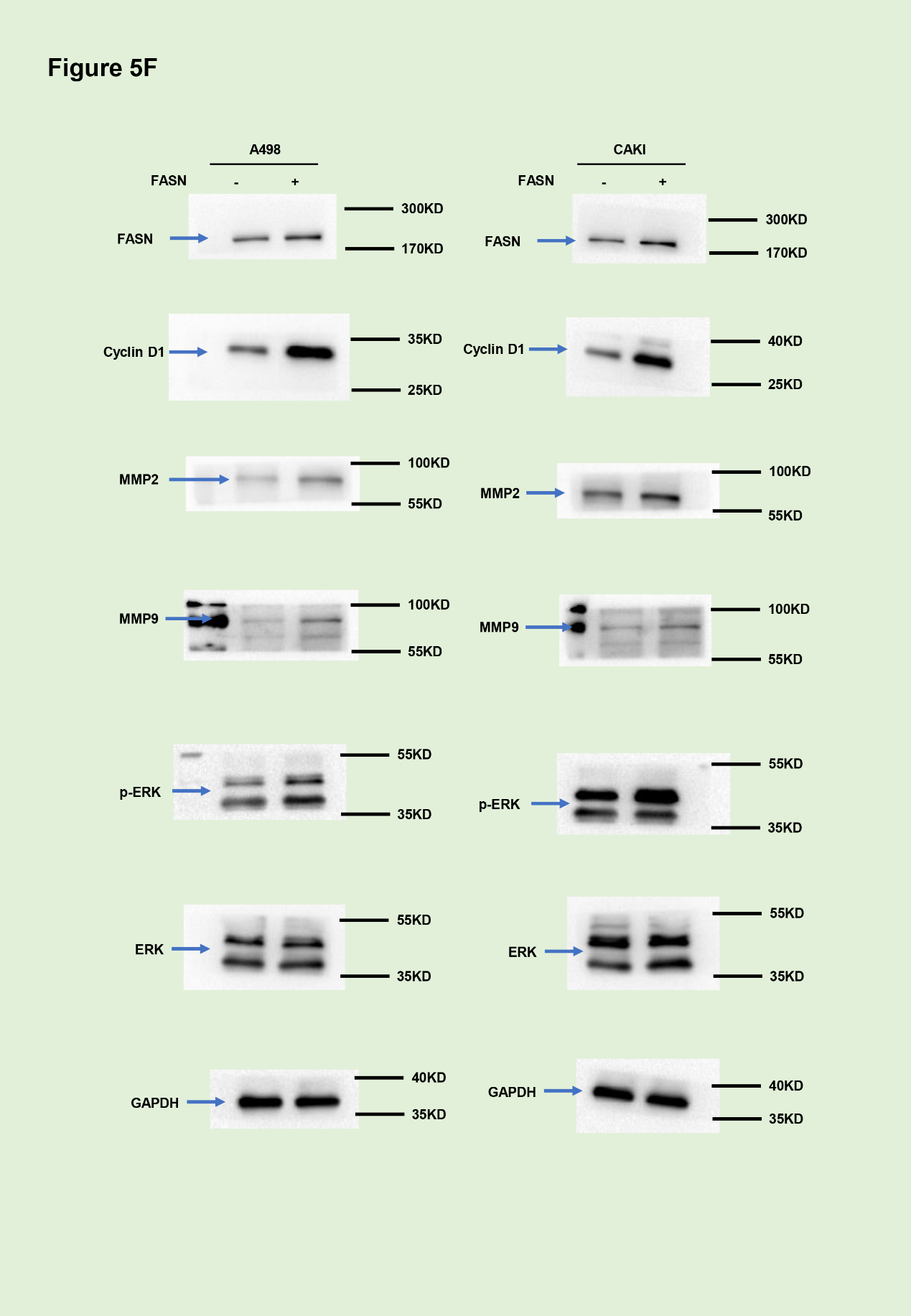

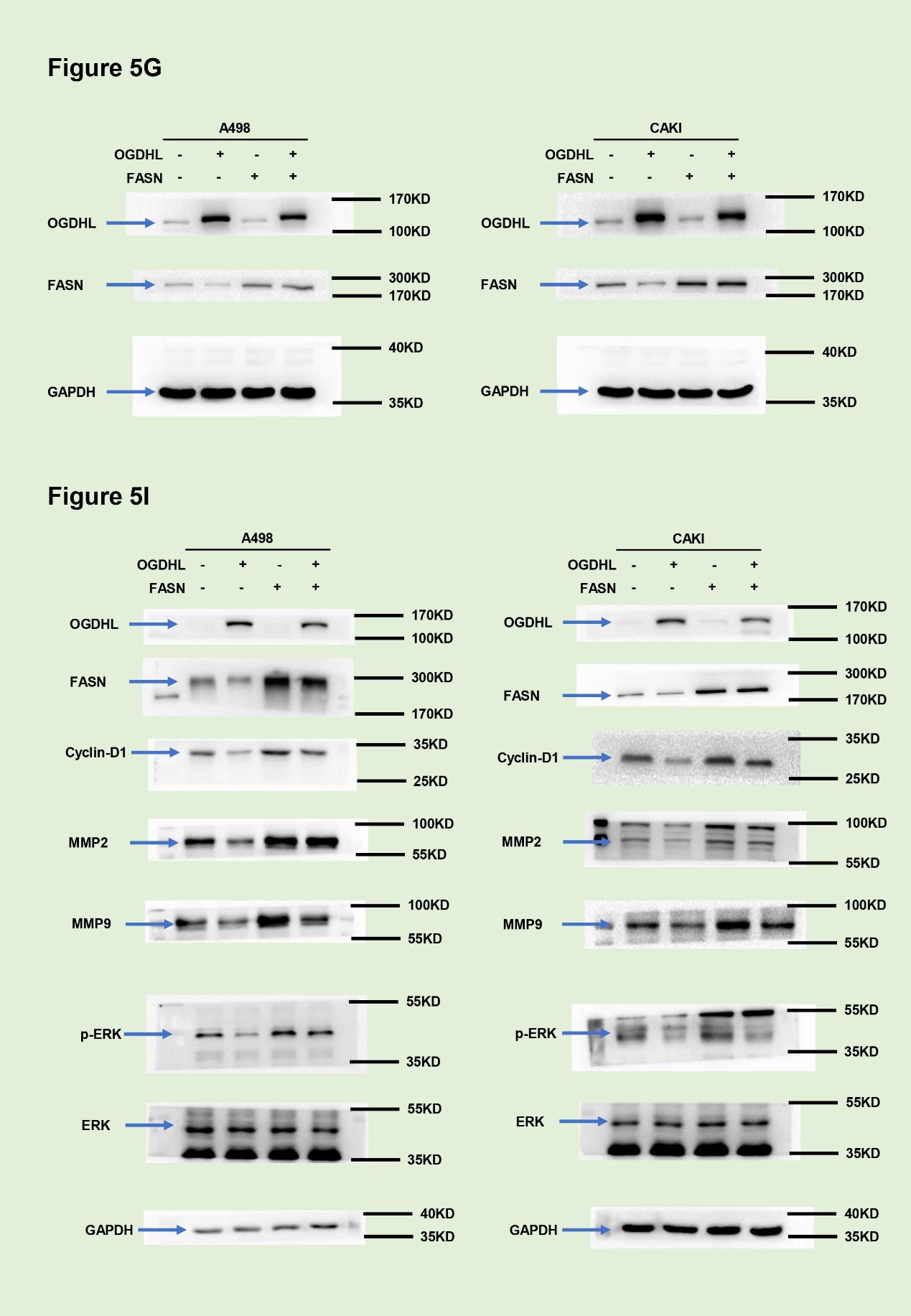

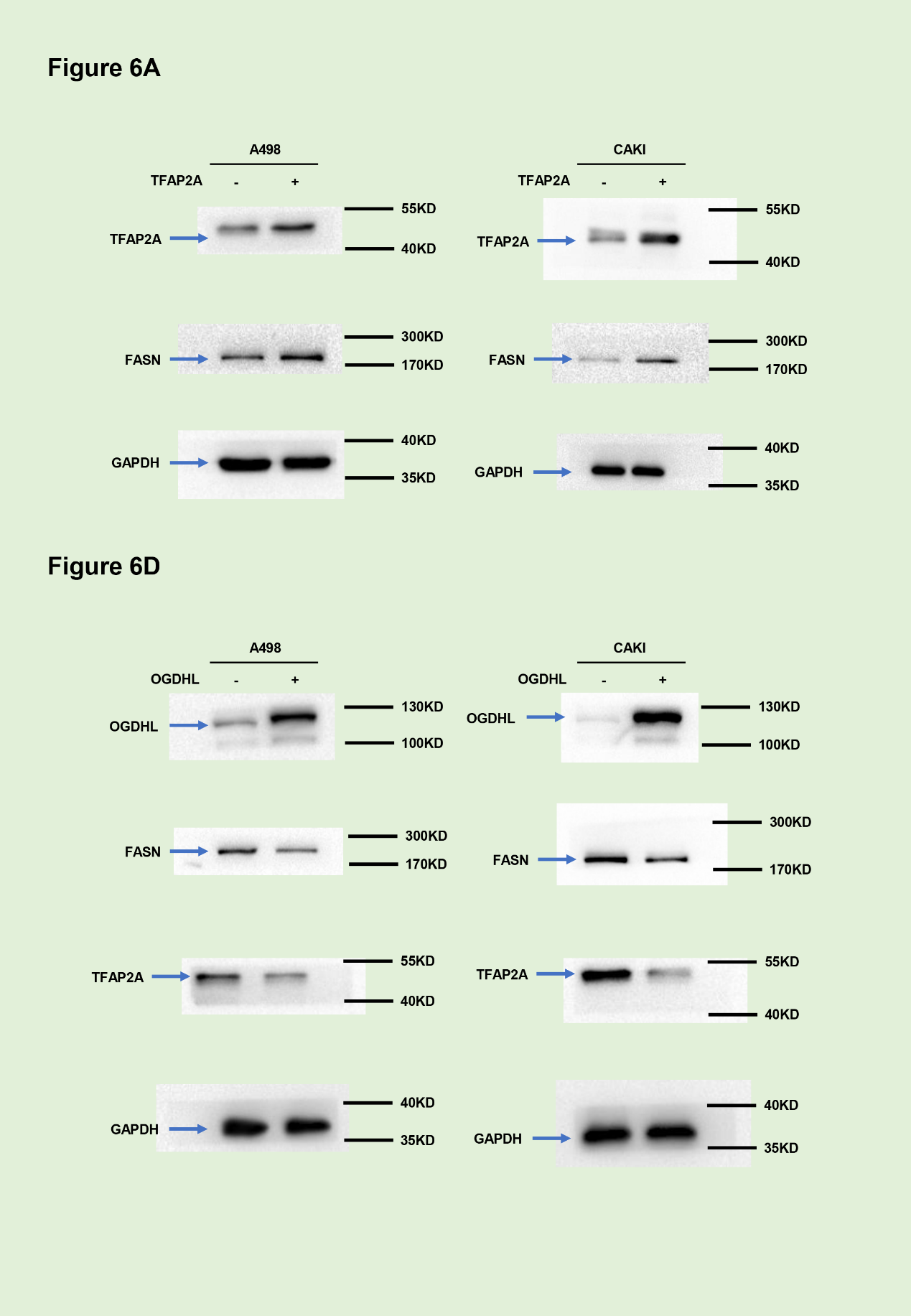

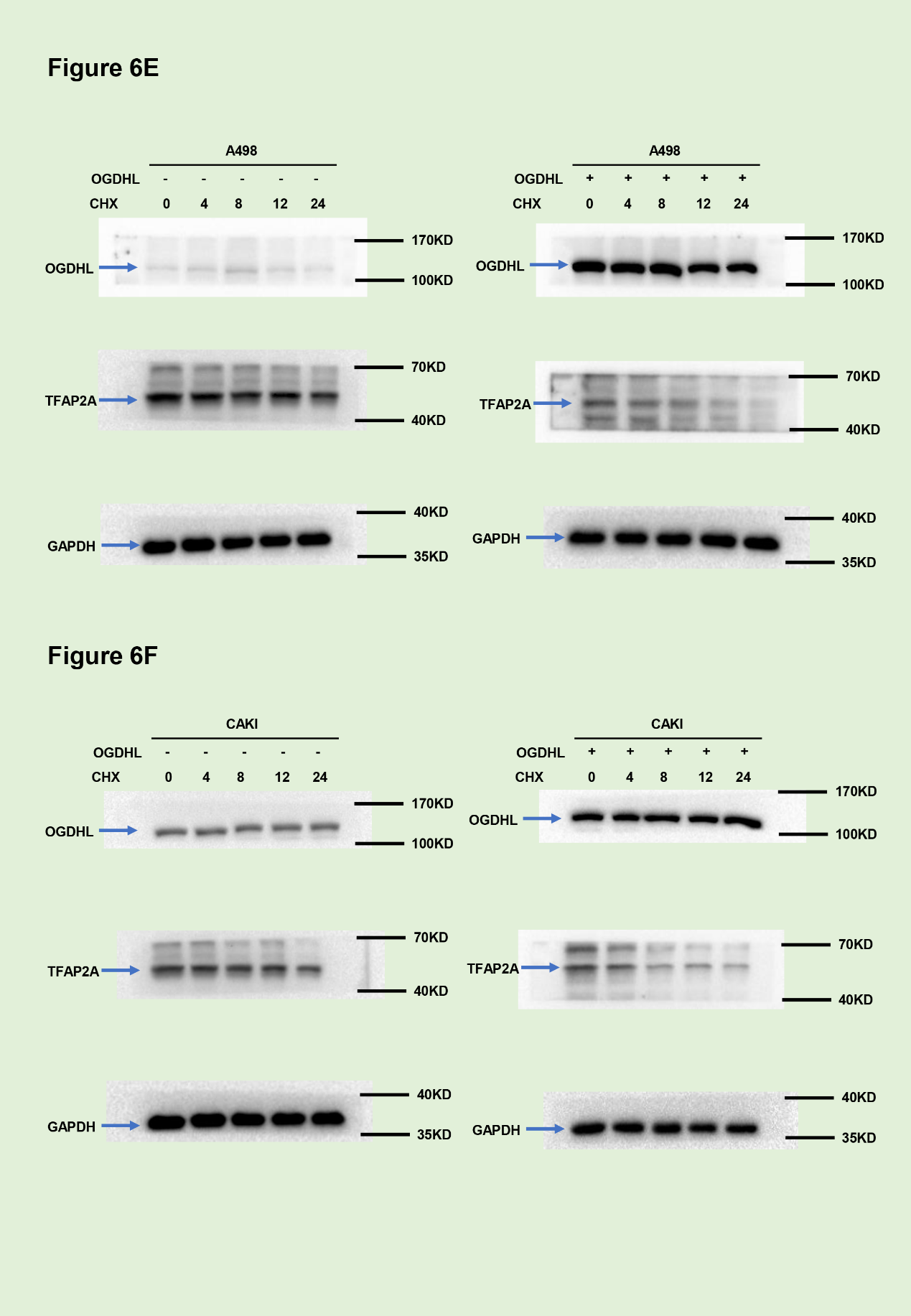

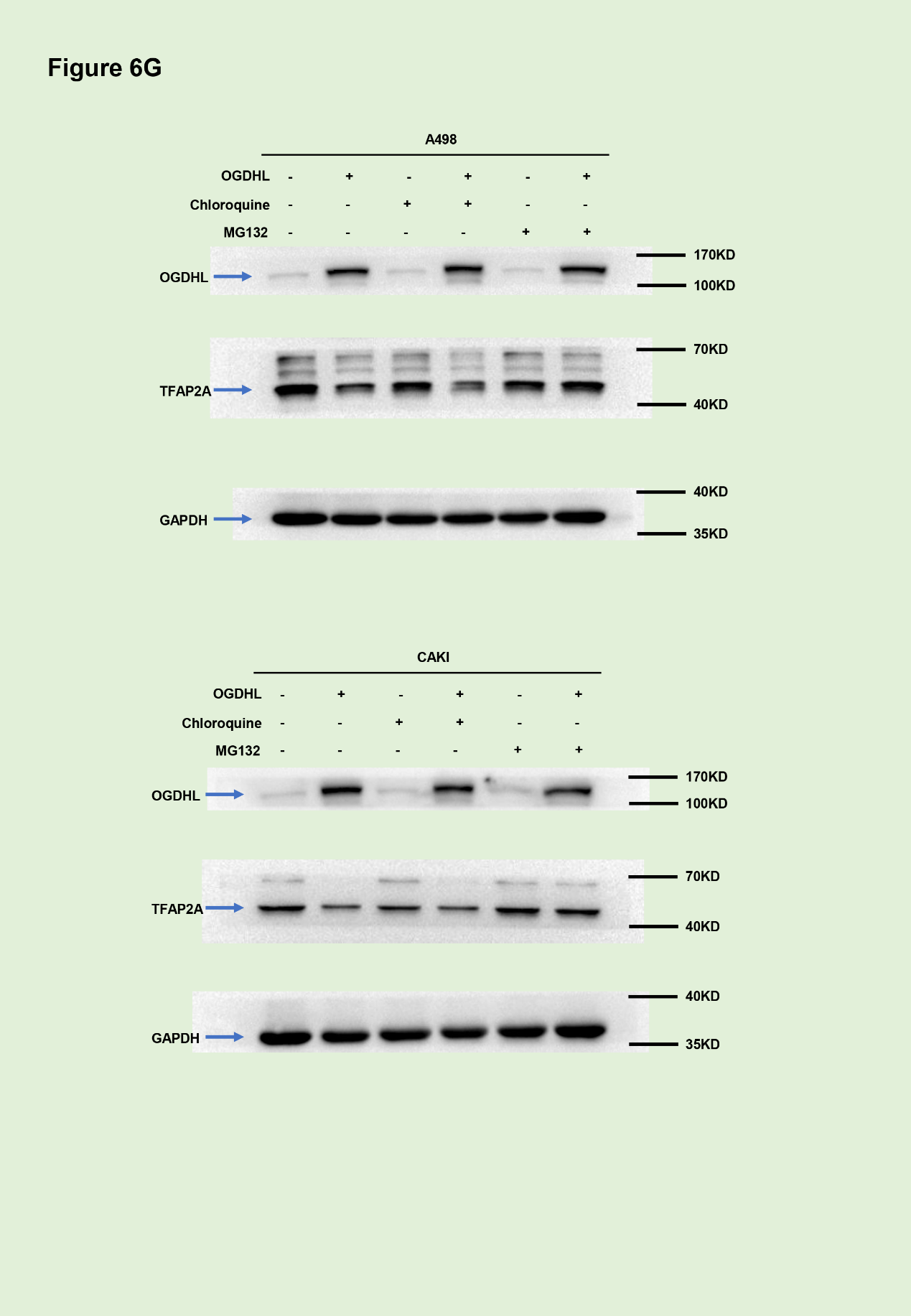

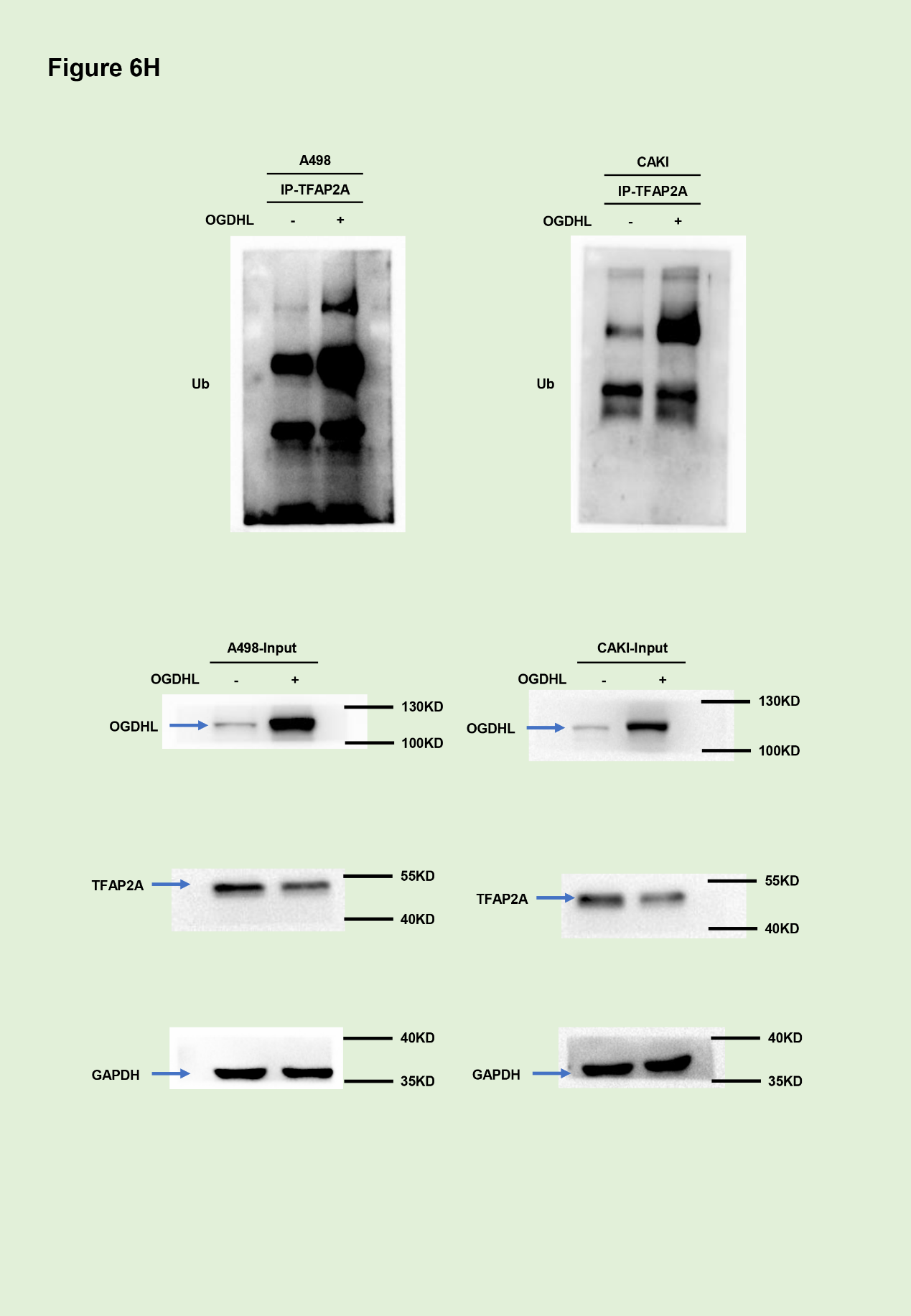

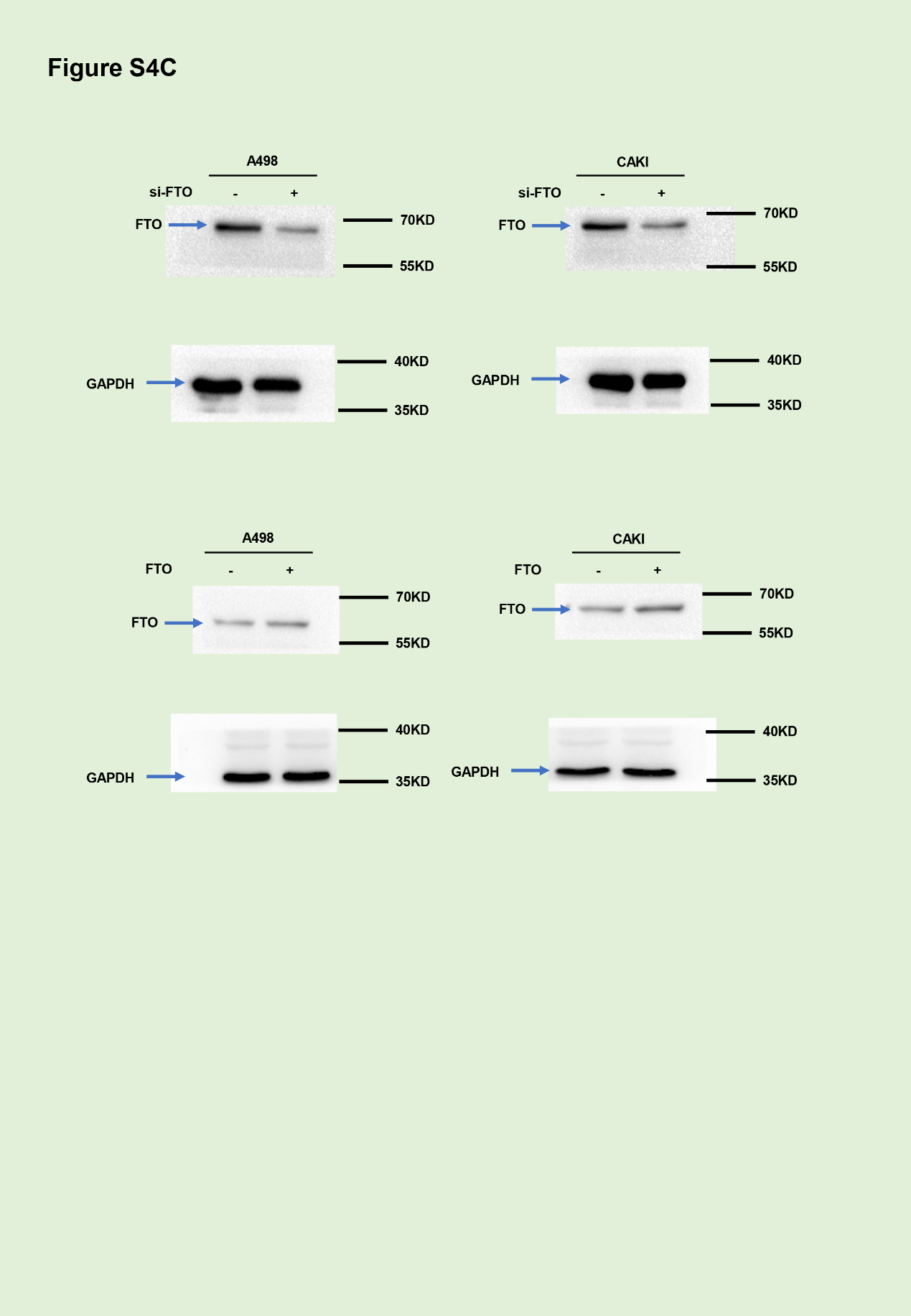

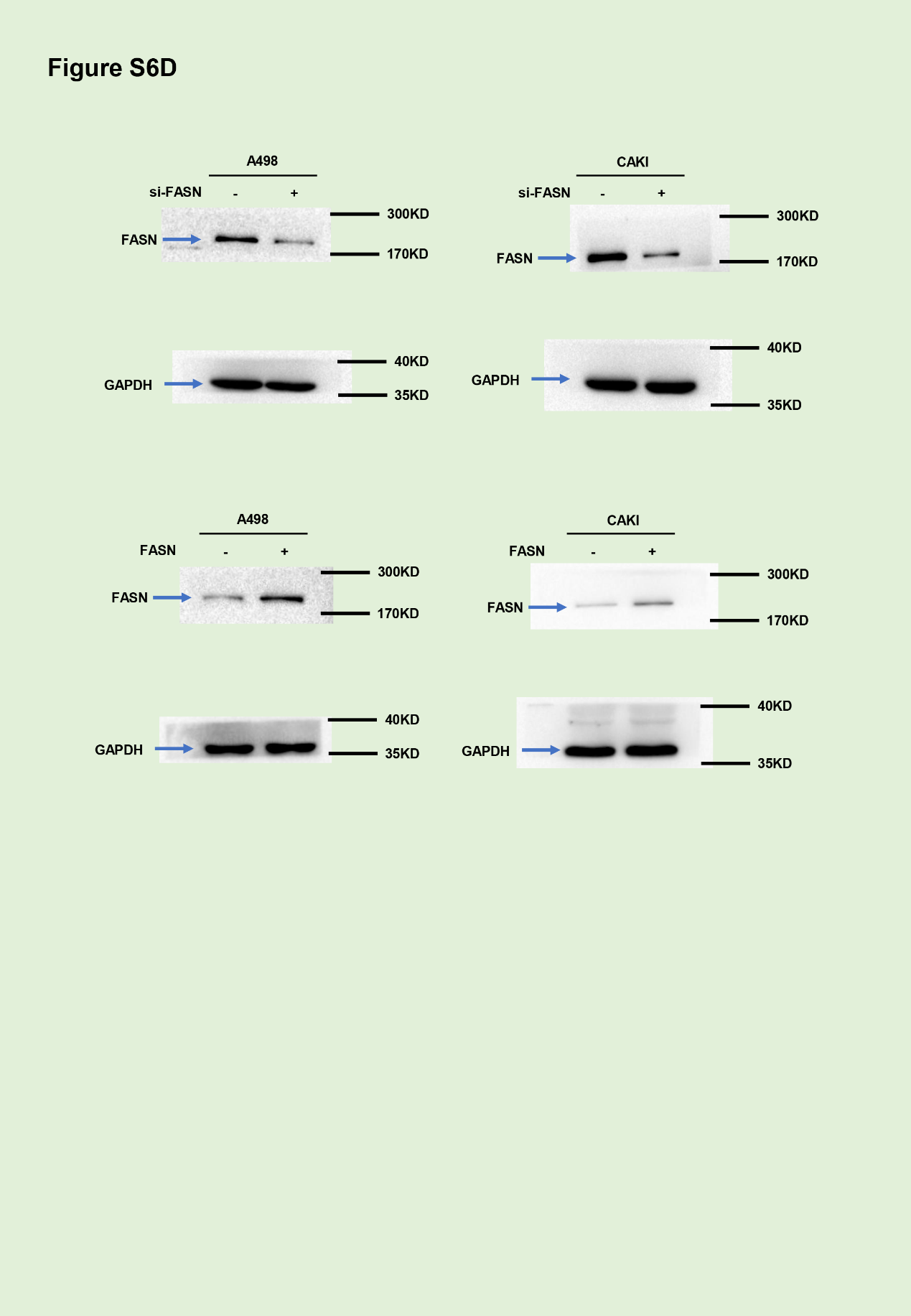

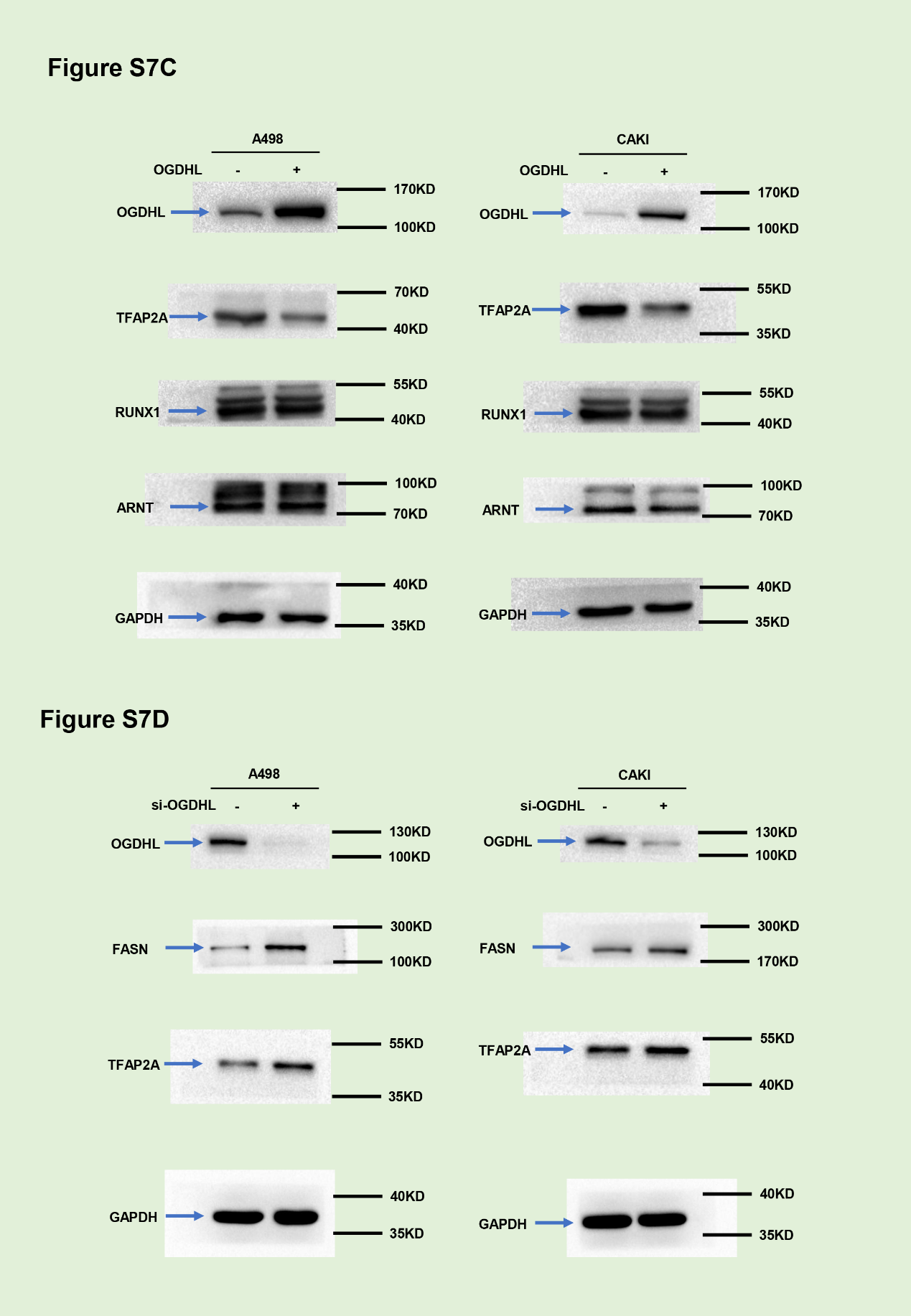

Supplement: Supplementary file 3 — Original Data File [file 41419_2023_6090_MOESM3_ESM.docx]
