# Supplementary material for: The m6A modification-mediated OGDHL exerts a tumor suppressor role in ccRCC by downregulating FASN to inhibit lipid synthesis and ERK signaling
Source: Cell Death Dis. 2023 Aug 25;14(8):560. doi: 10.1038/s41419-023-06090-7 (PMC10457380; doi:10.1038/s41419-023-06090-7)
Supplement: Supplementary file 2 [file 41419_2023_6090_MOESM2_ESM.docx]

**Supplementary figures**

**
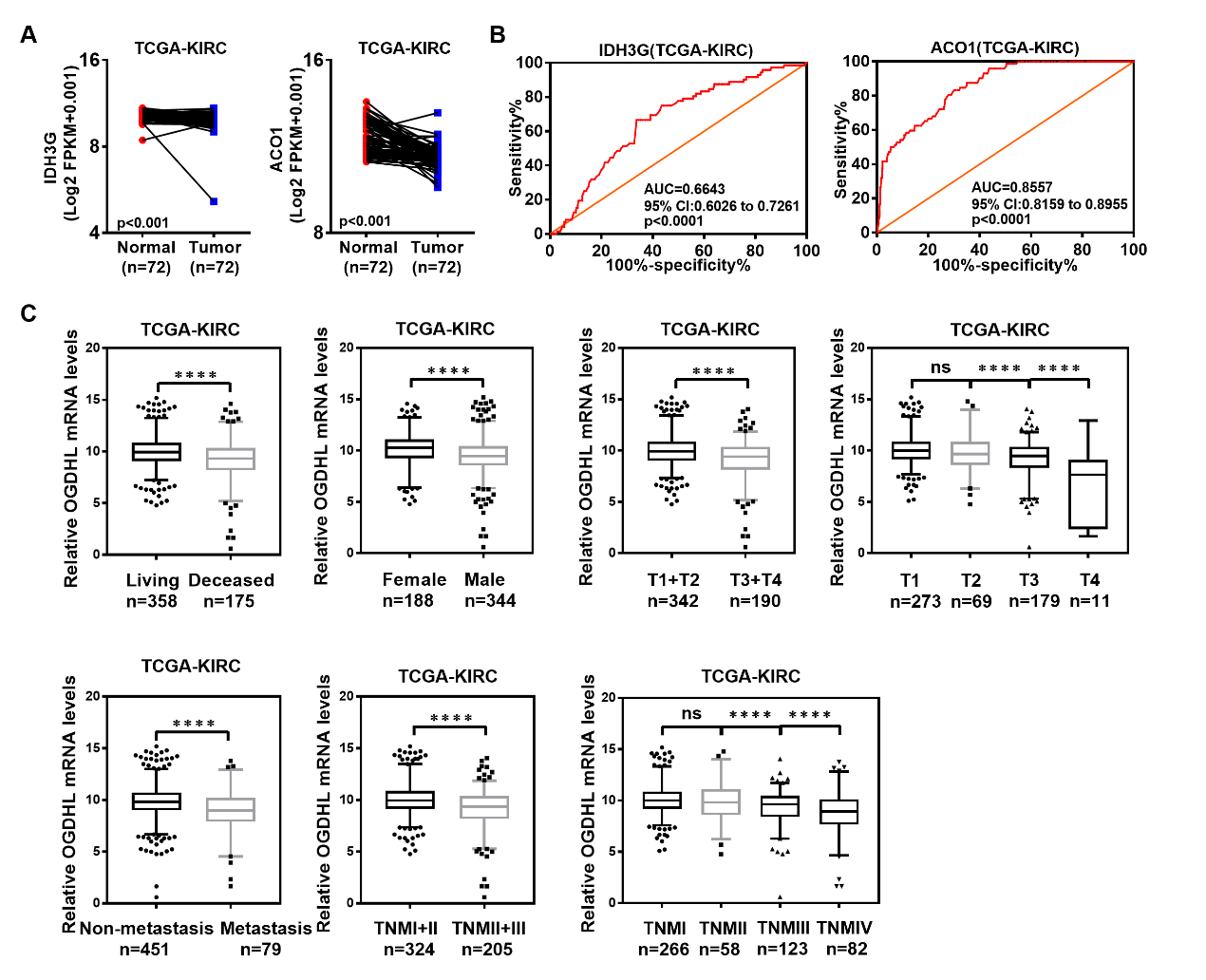
**

**Supplementary Figure S1. OGDHL is significantly downregulated in ccRCC and associated with poor patient outcomes**

**A.** The mRNA expression of IDH3G and ACO1 in 72 ccRCC tissues and their paired paracancerous tissues in the TCGA database. Paired samples t-test, p<0.05 was considered statistically significant.

**B.** ROC curves were drawn according to the expression levels of IDH3G and ACO1 in tumor samples and normal samples in the TCGA-KIRC database to evaluate the sensitivity and specificity of its diagnostic ability. ROC curve for IDH3G (AUC=0.6643 95% CI: 0.6026 to 0.7261; p < 0.0001), ROC curve for ACO1 (AUC=0.8557 95% CI: 0.8159 to 0.8955; p < 0.0001)

**C.** The correlations between OGDHL expression and different clinicopathological parameters of ccRCC from the TCGA-KIRC database; t-test, **** p <0.0001, *** p <0.001, ** p <0.01, and * p <0.05; ns, p≥0.05 (Independent-Samples t-test for statistics).


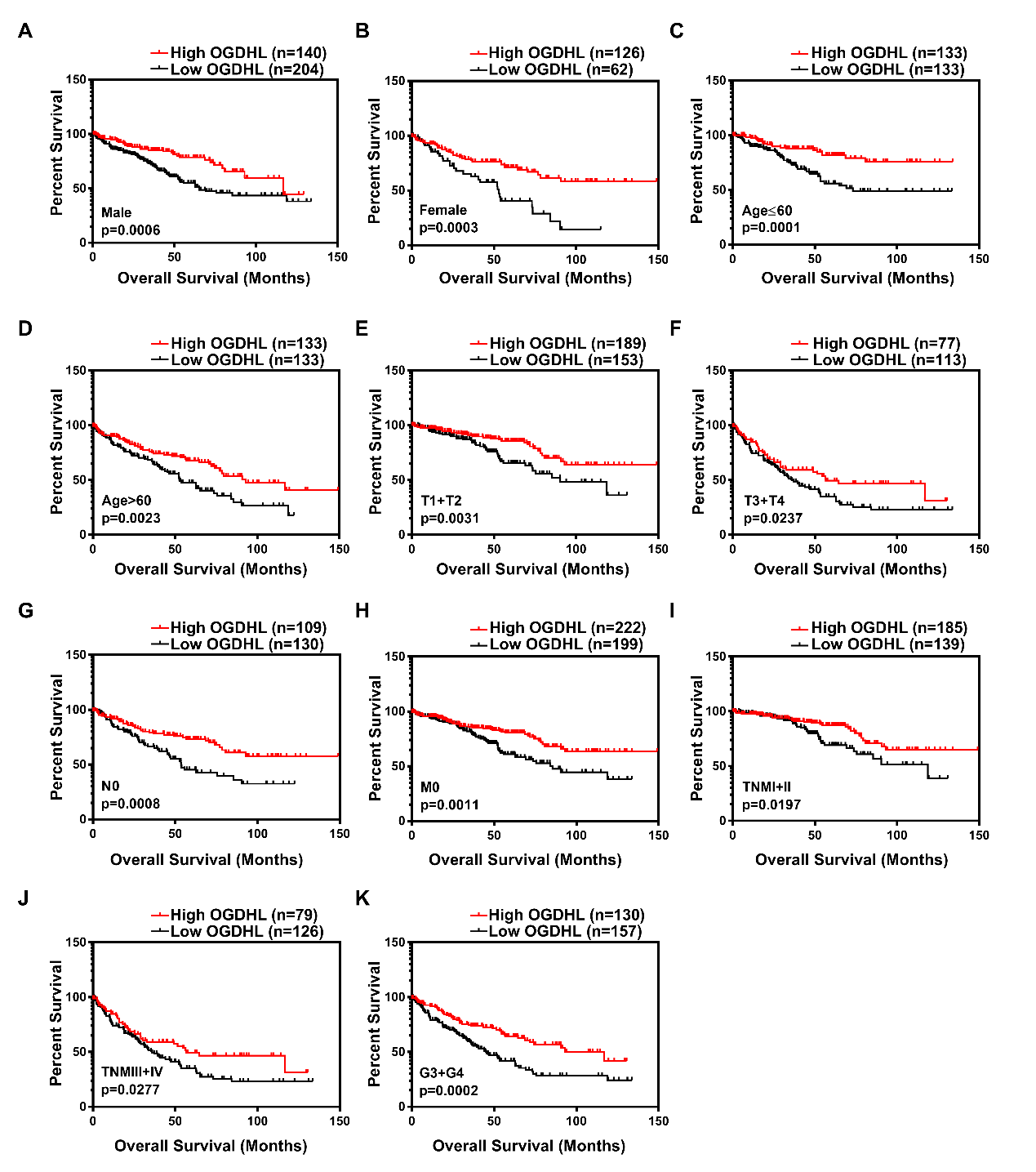


**Supplementary Figure S2. Low expression of OGDHL is associated with poor overall survival in patients with ccRCC.**

**A-K.** Kaplan-Meier analysis was performed according to different ccRCC clinical parameters to obtain (A-K) OS curves. The p value is obtained through Log-rank (Mantel-Cox) test. (A) Male. (B) Female. (C) Age ≤ 60. (D) Age > 60. (E) T1+T2 stage. (F) T3+T4 stage. (G) No lymph node metastasis. (H) Non-metastasis. (I) TNM I+ II. (J) TNM III+ IV stage. (K) G3+G4 stage.


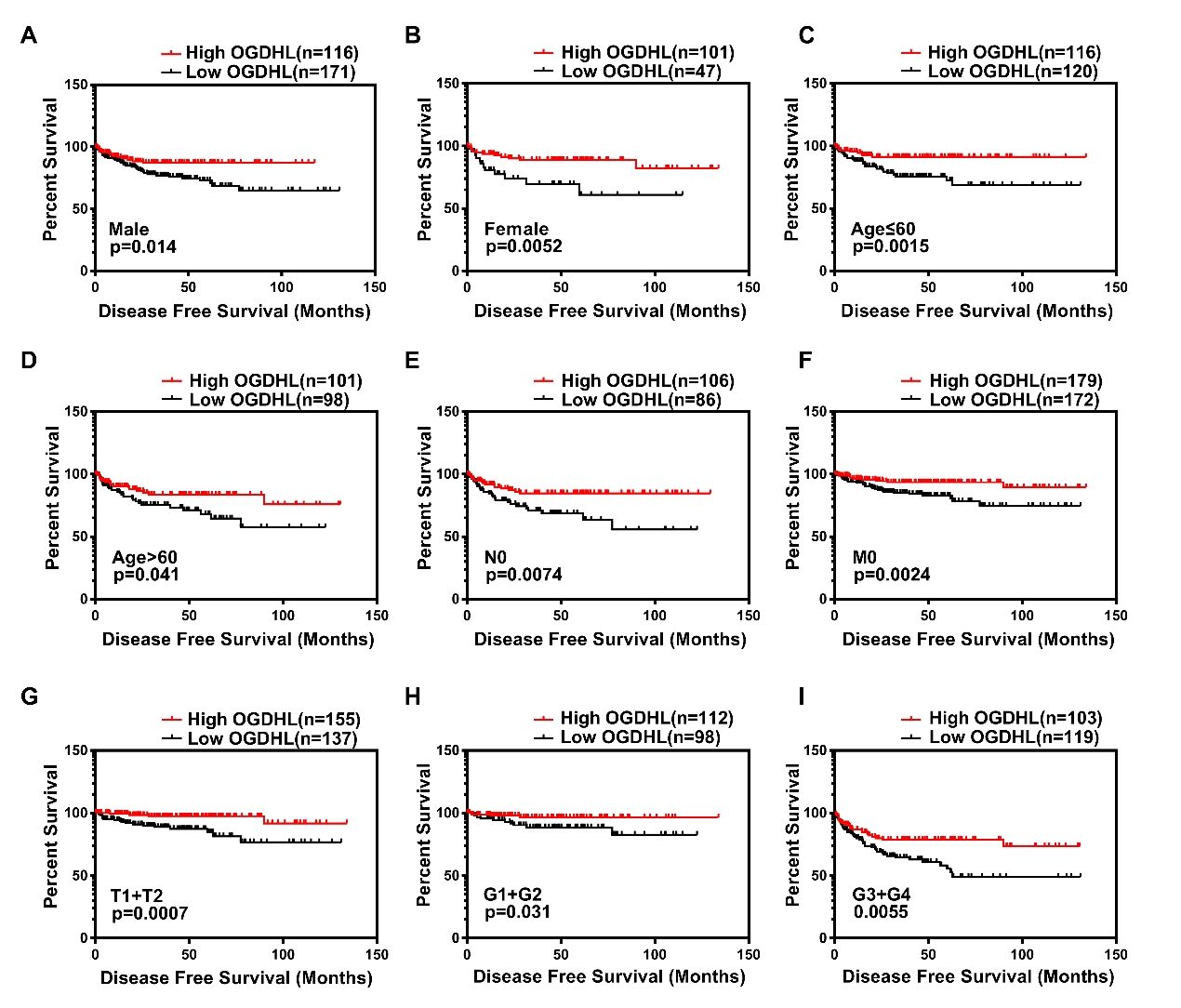


**Supplementary Figure S3. Low expression of OGDHL is associated with** **poor disease-free survival in patients with ccRCC.**

**A-I.** Kaplan-Meier analysis was performed according to different ccRCC clinical parameters to obtain (A-I) disease-free survival curves. The p value is obtained through Log-rank (Mantel-Cox) test. (A) Male. (B) Female. (C) Age ≤ 60. (D) Age > 60. (E) No lymph node metastasis. (F) Non-metastasis. (G) T1+T2 stage. (H) G1+G2 stage.

(I) G3+G4 stage.


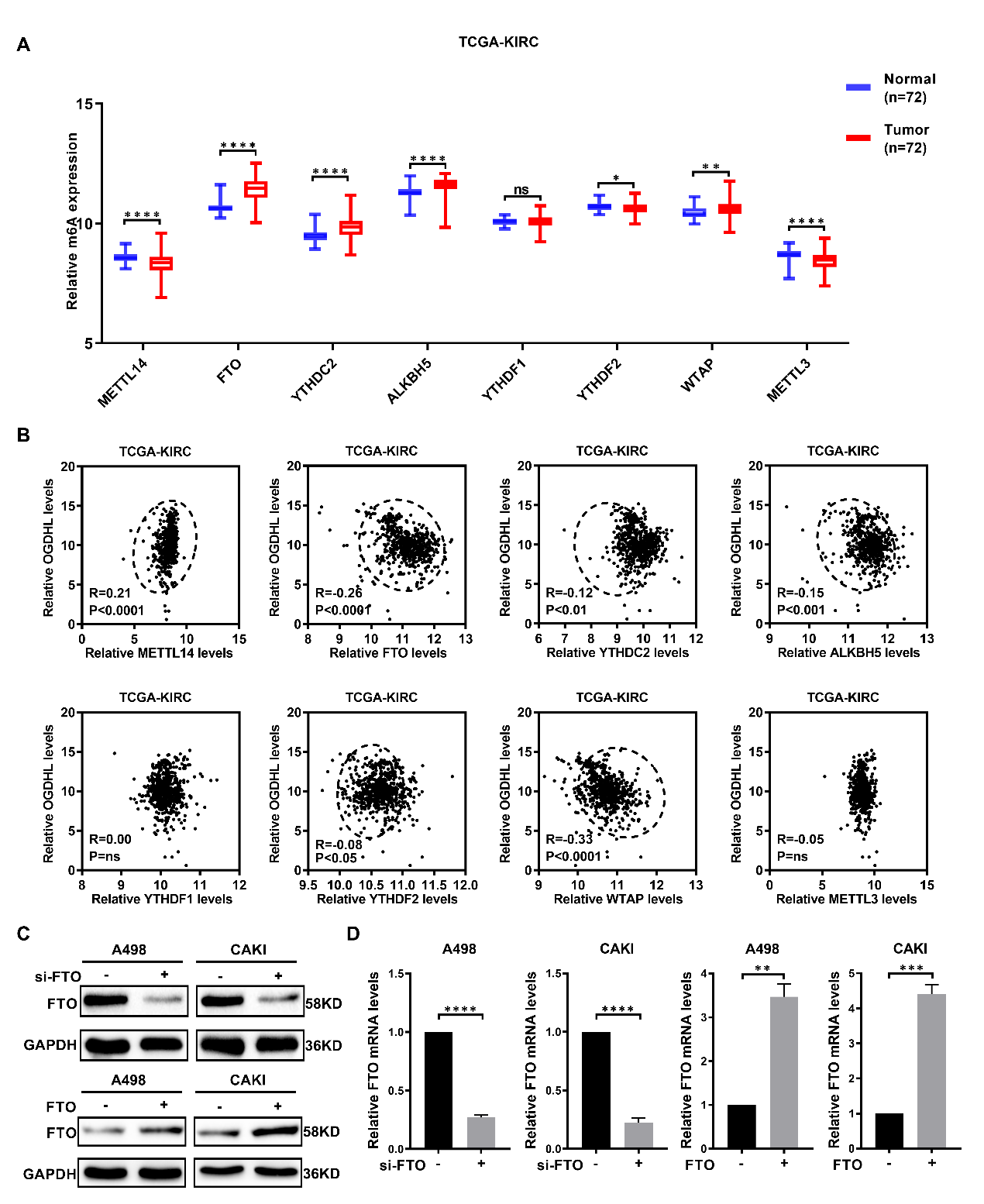


**Supplementary Figure S4.** **FTO-mediated m6A modification of OGDHL mRNA in ccRCC**

**A.** mRNA levels of m6a-regulated enzymes in 72 pairs of ccRCC and adjacent normal tissues from the TCGA-KIRC database.

**B.** The linear correlation curve between m6a-regulated enzymes and OGDHL based on the data of TCGA-KIRC database (R is the Pearson correlation coefficient).

**C.** Protein levels of FTO knockdown and overexpression in ccRCC cell lines were verified by western blot analysis.

**D.** The mRNA levels of FTO knockdown and overexpression in ccRCC cell lines were validated by qRT-PCT.


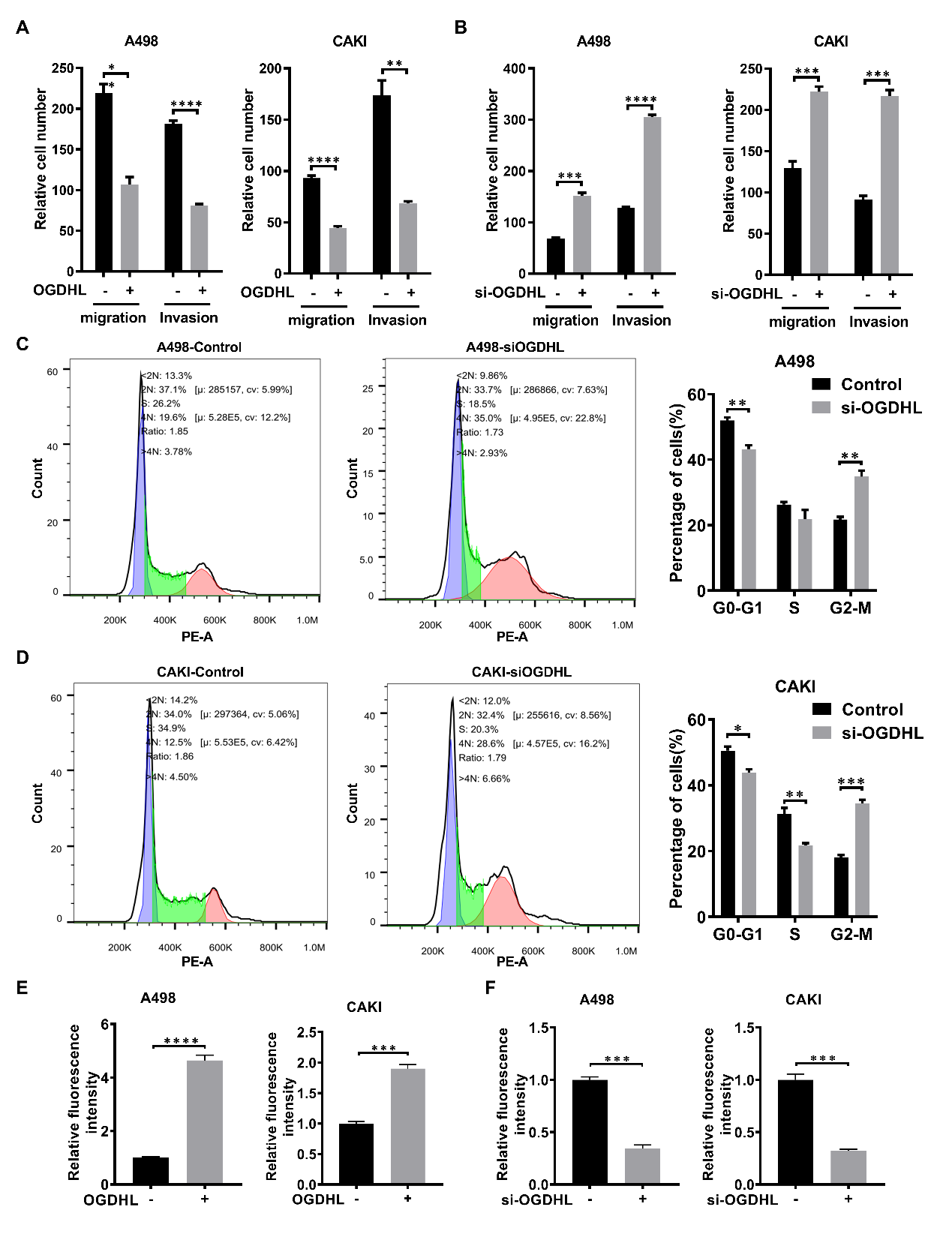


**Supplementary Figure S5. OGDHL inhibits the progress of ccRCC in vitro**

**A.** Quantitative analysis of Transwell results of OGDHL-overexpressing ccRCC cell lines.

**B**. Quantitative analysis of Transwell results of OGDHL knockdown ccRCC cell lines.

**C-D.** Flow cytometric analysis was performed on OGDHL knockdown and control ccRCC cell lines. Representative images and quantification of results are presented.

**E.** Quantitative analysis of Tunel fluorescence staining of OGDHL overexpressing and control ccRCC cell lines.

**F.** Quantitative analysis of Tunel fluorescence staining of OGDHL knockdown and control ccRCC cell lines.


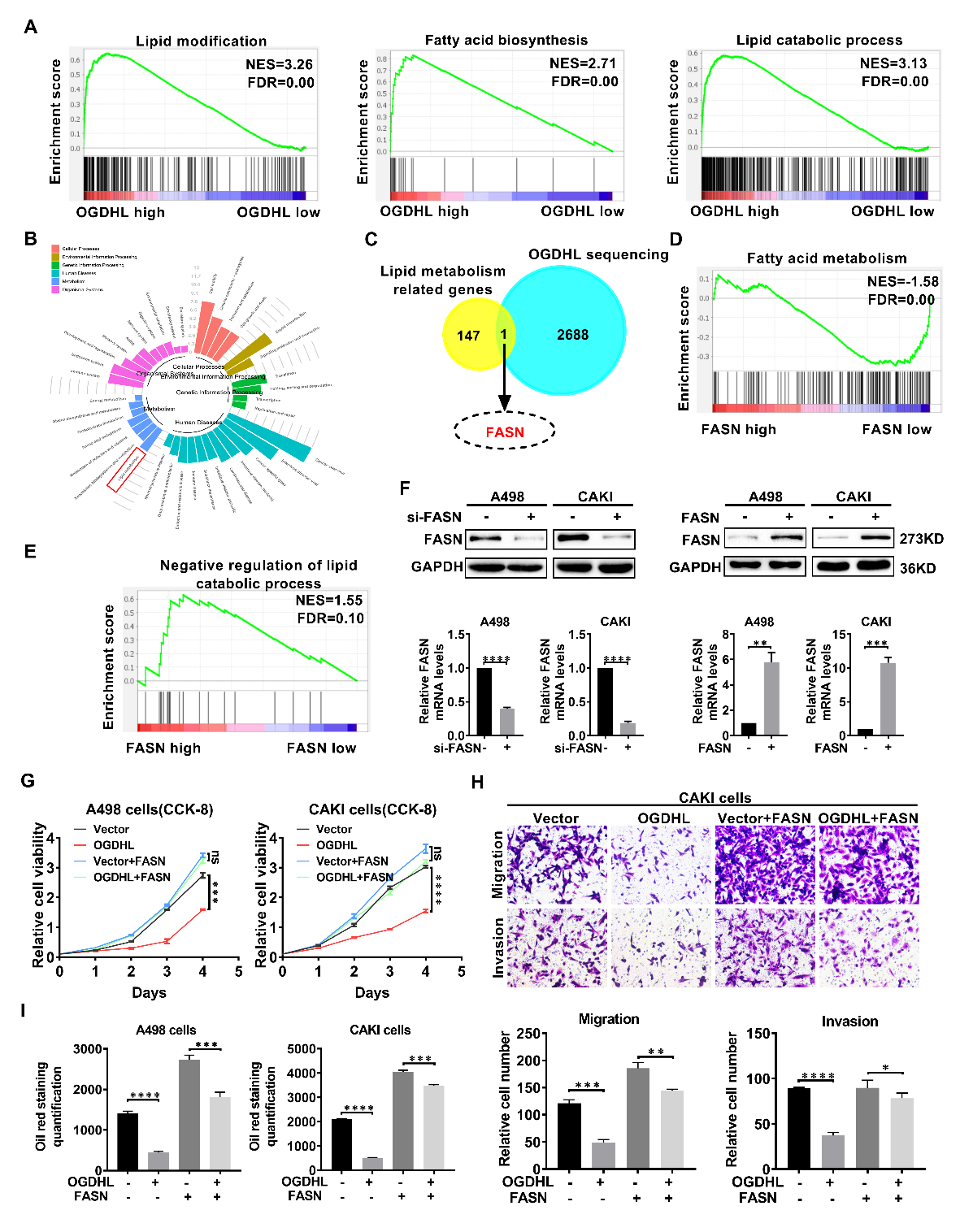


**Supplementary Figure S6.** **Up-regulated FASN-promoted lipid accumulation is a key link in the promotion of ccRCC by underexpressed OGDHL**

**A.** The GSEA correlation charts based on the TCGA-KIRC database with OGDHL expression as the premise for screening. FDR <25% and p <0.05 was considered statistically significant.

**B.** KEGG pathway analysis of whole transcriptome sequencing.

**C.** FASN was obtained by screening the differential gene set of OGDHL overexpressed whole transcriptome sequencing and the lipid metabolism gene set in the Oncomine database.

**D-E.** The GSEA correlation charts based on the TCGA-KIRC database with FASN expression as the premise for screening. FDR <25% and p <0.05 was considered statistically significant.

**F.** Western blot analysis and qRT-PCR were used to verify the knockdown and overexpression of FASN in ccRCC cell lines.

**G.** Cell growth curves based on CCK8 assays are shown for stable cell lines; t-test, **** p <0.0001, *** p <0.001, ** p <0.01, and * p <0.05 (Independent-Samples t-test for statistics).

**H.** The results of the transwell assay of the migration and invasion of stable cell lines; t-test, **** p <0.0001, *** p <0.001, ** p <0.01, and * p <0.05 (Independent-Samples t-test for statistics).

**I.** Quantitative analysis of Oil Red O staining; t-test, **** p <0.0001, *** p <0.001, ** p <0.01, and * p <0.05 (Independent-Samples t-test for statistics).


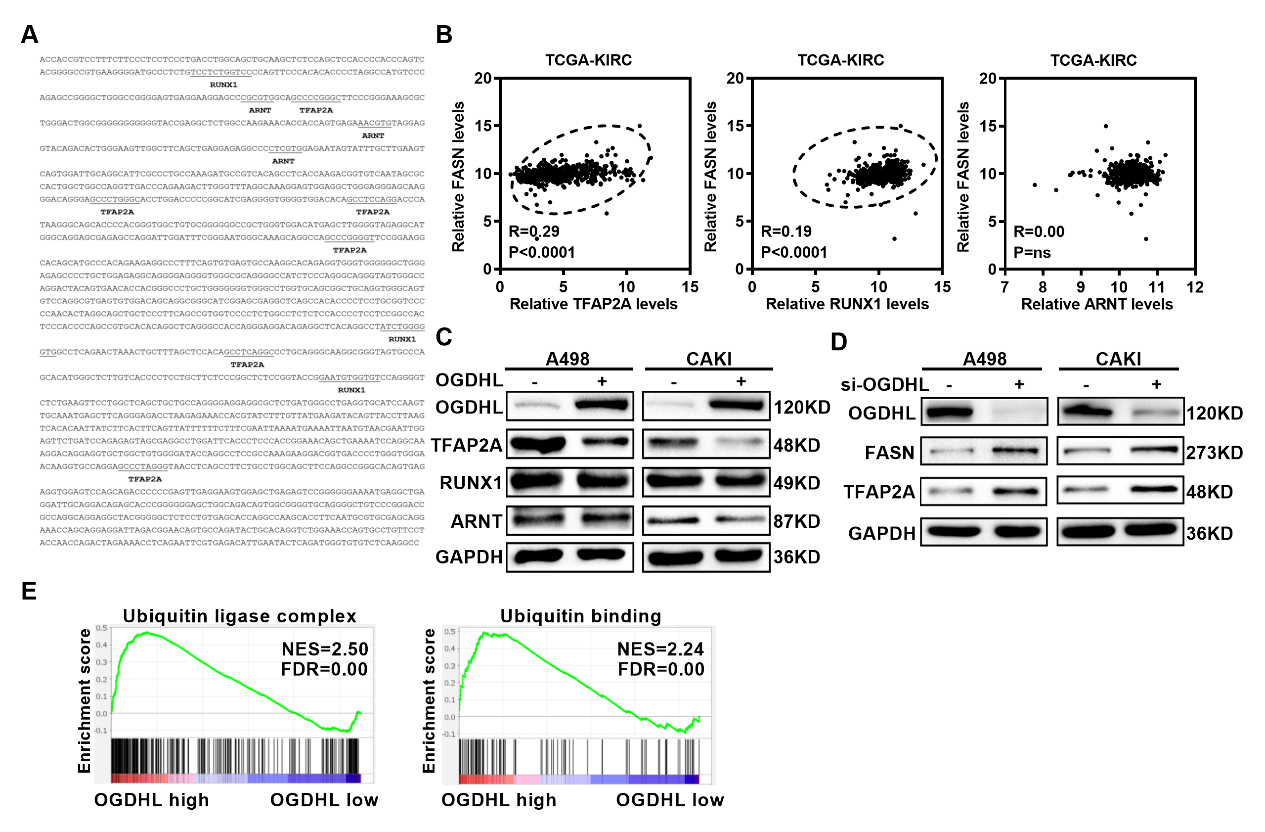


**Supplementary Figure S7. Regulation of FASN-induced biological effects by OGDHL is mediated by TFAP2A transcriptional regulation**

**A.** Potential transcription factors and possible binding sites for FASN were predicted using the Jaspar program database.

**B.** Linear correlation curves between predicted transcription factors (TFAP2A, RUNX1, ARNT) and FASN based on TCGA-KIRC database data (R is the Pearson correlation coefficient).

**C.** Western blot analysis was used to detect the protein levels of TFAP2A, RUNX1 and ARNT in OGDHL-overexpressing ccRCC cell lines.

**D.** The protein levels of FASN and TFAP2A in OGDHL knockdown ccRCC cell lines were detected by western blot analysis.

**E.** The GSEA correlation charts based on the TCGA-KIRC database with OGDHL expression as the premise for screening. FDR <25% and p <0.05 was considered statistically significant.
